# A smartphone app to reduce excessive alcohol consumption: Identifying the effectiveness of intervention components in a factorial randomised control trial

**DOI:** 10.1038/s41598-018-22420-8

**Published:** 2018-03-12

**Authors:** David Crane, Claire Garnett, Susan Michie, Robert West, Jamie Brown

**Affiliations:** 1 Department of Clinical, Educational and Health Psychology, University College London, London, UK; 2 Department of Behavioural Science and Health, University College London, London, UK

## Abstract

Our aim was to evaluate intervention components of an alcohol reduction app: Drink Less. Excessive drinkers (AUDIT> =8) were recruited to test enhanced versus minimal (reduced functionality) versions of five app modules in a 2^5^ factorial trial. Modules were: Self-monitoring and Feedback, Action Planning, Identity Change, Normative Feedback, and Cognitive Bias Re-training. Outcome measures were: change in weekly alcohol consumption (primary); full AUDIT score, app usage, app usability (secondary). Main effects and two-way interactions were assessed by ANOVA using intention-to-treat. A total of 672 study participants were included. There were no significant main effects of the intervention modules on change in weekly alcohol consumption or AUDIT score. There were two-way interactions between enhanced Normative Feedback and Cognitive Bias Re-training on weekly alcohol consumption (F = 4.68, p = 0.03) and between enhanced Self-monitoring and Feedback and Action Planning on AUDIT score (F = 5.82, p = 0.02). Enhanced Self-monitoring and Feedback was used significantly more often and rated significantly more positively for helpfulness, satisfaction and recommendation to others than the minimal version. To conclude, in an evaluation of the Drink Less smartphone application, the combination of enhanced Normative Feedback and Cognitive Bias Re-training and enhanced Self-monitoring and Feedback and Action Planning yielded improvements in alcohol-related outcomes after 4-weeks.

## Introduction

Reducing excessive alcohol consumption is a public health priority^1^. Face-to-face interventions appear to be both effective^2^ and cost-effective^3,4^ but are not widely offered by physicians or sought by patients in countries such as the UK and US^5,6^. Digital behaviour change interventions (DBCIs) may overcome the cost, time and training barriers experienced when delivering interventions in person^7–9^. Meta-analyses of alcohol DBCIs have consistently found small reductions in consumption amongst the general public and students (e.g.^10–12^). A Cochrane review found that DBCIs reduced alcohol consumption by 23.6 grams of alcohol per week (equivalent to 2.95 UK units or 1.69 US standard drinks) more than control groups receiving alcohol-related information, usual care, or baseline measures only^13^. Effect sizes were greatest for the trials that conducted follow-up at two to three months (−43.3 g/week, range: −73.2 to −13.4, p < 0.01), at follow-ups longer than three months the effect size of the intervention reduced to a mean reduction of −11.5 g/week (range: −16.3 to −6.7, p < 0.001)^13^.

The vast majority of alcohol DBCIs have been provided on websites^13^. Smartphone applications (apps) provide a new way to support people who are attempting to reduce their alcohol consumption; however, most alcohol reduction apps appear to be developed without explicit reference to scientific evidence or theory^14^. Whilst there have been numerous trials of text messaging for alcohol reduction (e.g.^15,16^) there has been little evaluation of the effectiveness of apps. Two apps have demonstrated effectiveness in pilot studies^17,18^. One app was effective in reducing the number of risky drinking days in dependent drinkers (risky drinking days were defined as more than four drinks for men or three drinks for women in a two-hour period)^19^. Another app, which appears to the only published trial of an app aimed at hazardous and harmful drinkers, found no reduction in consumption in the experimental group relative to controls for university students in Sweden^20^. The lack of evidence for the effectiveness of alcohol reduction apps and the tendency of publicly available apps to be developed without use of theory or evidence highlights the need for the rigorous development and evaluation of new alcohol app interventions.

The app reported in this study, *Drink Less*, consisted of modules containing multiple behaviour change techniques (see^21^ for details). The enhanced (experimental) version of each module contained the ‘active ingredients’ hypothesised to be effective at reducing excessive alcohol consumption. The minimal (control) versions of each module were designed to provide some support to participants for ethical reasons, whilst excluding the potentially active ingredients of the enhanced version. The following five modules were selected as high priority for experimental manipulation in a factorial design: *Normative Feedback*; *Cognitive Bias Re-training*; *Self-monitoring and Feedback*; *Action Planning*, and *Identity Change*. Four primary sources of evidence informed the selection of these modules: i) a study which examined the behaviour change techniques (BCTs) used in face-to-face alcohol interventions^22^; ii) a systematic review of the evidence of the effectiveness of DBCIs in reducing excessive alcohol consumption^13^; iii), a formal consensus-building study conducted with experts in alcohol or behaviour change to identify the BCTs thought most likely to be effective in reducing alcohol consumption in an app^23^; and iv), a content analysis of the type and prevalence of BCTs in existing popular alcohol reduction apps^14^. The reasons for the selection of each module is summarised in Supplementary File 1.

Modular-based DBCIs require an evaluation design that can assess the effectiveness of individual modules. Randomised control trials are effective at evaluating an intervention as a whole, but are not able to evaluate the independent or interactive effect of intervention components^24^. Factorial RCTs, guided by the Multiphase Optimization Strategy (MOST), allow multiple variables and their interactions to be evaluated simultaneously, without requiring a large sample size^25^. Sample sizes are reduced in factorial designs because participants are, in effect, assigned to multiple conditions. Analysis is then performed between participants who received the experimental and control version for one particular condition. For example, in our trial participants in groups 1–16 received Normative Feedback enhanced and were compared against participants in groups 17–32 who received Normative Feedback minimal (see Supplementary File 2 for the experimental group matrix). Factorial trials, therefore, need to be powered to detect an effect between conditions, whereas in a traditional RCT that conducted five experiments approximately five times as many participants would be required. This makes factorial RCTs highly suited for trials of a complex DBCI such as the one reported here, though the vast majority of existing health-related app evaluations still use a traditional RCT^26^.

The aim of this study was to evaluate the effectiveness of the five intervention modules at reducing excessive alcohol consumption and to investigate interactions between modules using a factorial randomised control trial.

### Research questions

1. What are the main effects of, and interactions between, each intervention module on:

- Change in weekly alcohol consumption
- Change in full AUDIT (Alcohol Use Disorders Identification Test) score
- App usage
- Usability ratings for the app

## Results

### Participants

**Figure 1:**
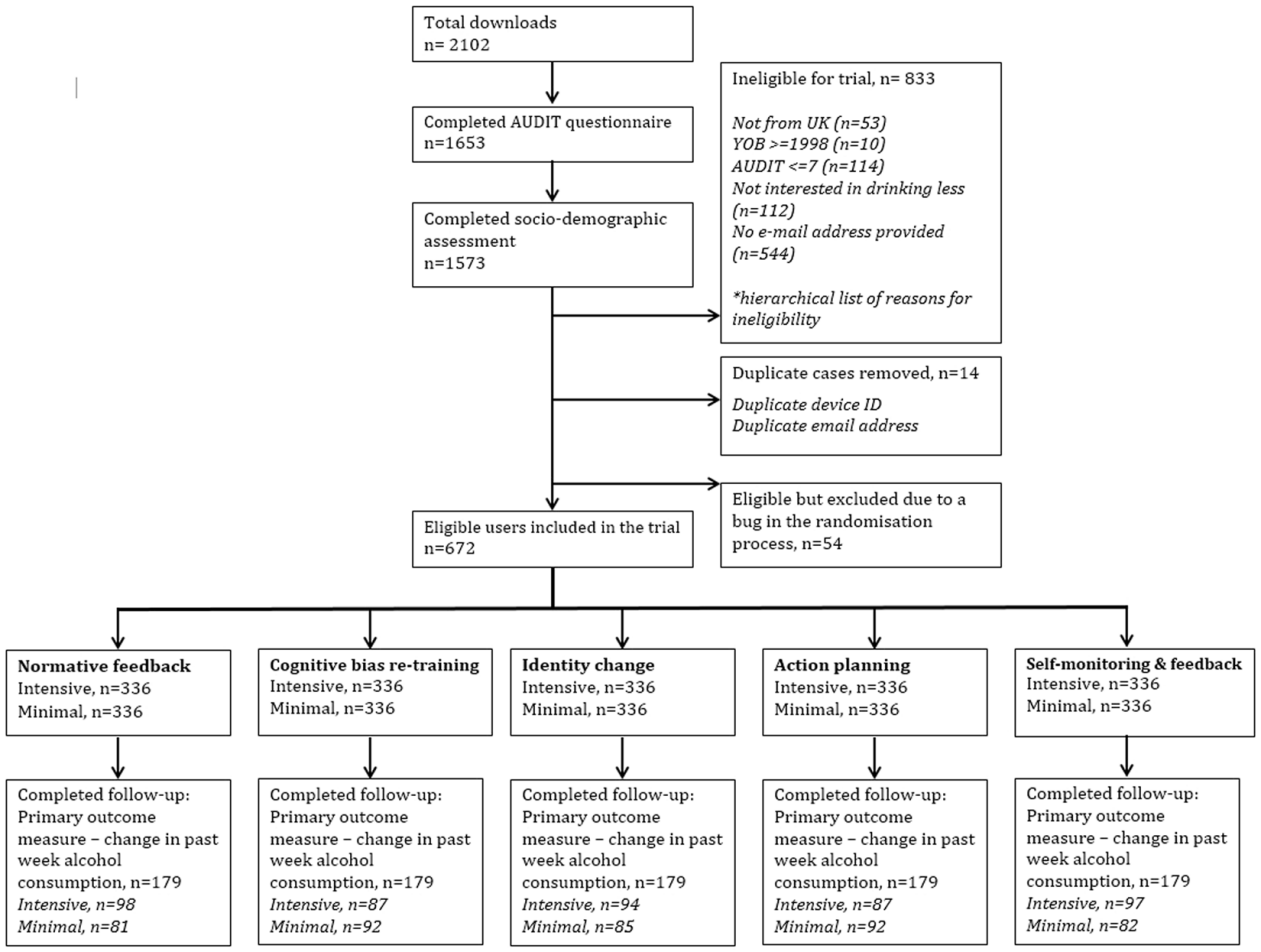
AUDIT = Alcohol Use Disorders Identification Test, YOB = Year of birth, intensive = intensive (experimental) version of the app, minimal = minimal (control) version of the app.

Recruitment began on 18^th^ May 2016 and ended on 10^th^ July 2016 when each of the 32 conditions had 21 eligible users after accounting for duplicate cases. Follow-up data were collected between 16^th^ June and 28^th^ August 2016. Of the 672 eligible users, 179 (27%) completed the primary outcome measure at follow-up. There were no significant differences in retention rate between versions of the intervention modules. Fig. 1 shows a flow chart of users from the trial.

### Baseline characteristics

Socio-demographic and drinking characteristics of participants are reported in Table 1. Participants’ mean age was 39.2 years, 56.1% were women, 95.2% were white, 72.0% had post-16 qualifications, 86.5% were employed and 24.6% were current smokers. Mean weekly alcohol consumption was 39.9 units, mean AUDIT score was 19.1 and mean AUDIT-C score was 9.4. Two-thirds of participants (66.7%) had an AUDIT score of 16 or above, indicating harmful drinking or drinkers at-risk of alcohol dependence.

**Table 1:** Participant characteristics at baseline: Mean (n), unless stated.

	All	Normative Feedback		Cognitive Bias Re-training		Self-monitoring and Feedback		Action Planning		Identity Change	
		Enh	Min	Enh	Min	Enh.	Min	Enh	Min	Enh	Min
Age (SD)	39.2 (10.92)	38.3* (10.14)	40.0* (11.60)	39.4 (10.92)	39.0 (10.93)	39.4 (11.46)	38.9 (10.36)	39.4 (11.44)	39.0 (10.39)	39.9 (11.02)	38.5 (10.78)
% Women	56.1 (377)	55.1 (185)	57.1 (192)	56.3 (189)	56.0 (188)	59.2 (199)	53.0 (178)	57.1 (192)	55.1 (185)	53.3 (179)	58.9 (198)
% White	95.2 (640)	95.5 (321)	94.9 (319)	95.2 (320)	95.2 (320)	94.6 (318)	95.8 (322)	95.2 (320)	95.2 (320)	95.2 (320)	95.2 (320)
% Post-16 qualifications	72.0 (484)	72.0 (242)	72.0 (242)	72.6 (244)	71.4 (240)	72.6 (244)	71.4 (240)	72.0 (242)	72.0 (242)	72.0 (242)	72.0 (242)
% Employed	86.5 (581)	86.9 (292)	86.0 (289)	87.5 (294)	85.4 (287)	83.6* (281)	89.3* (300)	83.0* (279)	89.9* (302)	86.9 (292)	86.0 (289)
% Current smokers	24.6 (165)	25.0 (84)	24.1 (81)	24.4 (82)	24.7 (83)	24.4 (82)	24.7 (83)	22.6 (76)	26.5 (89)	23.2 (78)	25.9 (87)
PWC Units (SD)	39.9 (27.34)	39.1 (25.97)	40.7 (28.66)	40.3 (28.23)	39.6 (26.45)	39.9 (27.09)	39.9 (27.63)	39.0 (26.46)	40.9 (28.20)	39.0 (26.62)	40.8 (28.05)
AUDIT score (SD)	19.1 (6.56)	19.2 (6.49)	18.9 (6.63)	19.2 (6.75)	18.9 (6.37)	18.9 (6.44)	19.2 (6.69)	19.0 (6.68)	19.1 (6.45)	19.0 (6.35)	19.2 (6.77)
AUDIT-C score (SD)	9.4 (1.85)	9.4 (1.77)	9.4 (1.92)	9.4 (1.95)	9.4 (1.74)	9.4 (1.86)	9.4 (1.84)	9.4 (1.78)	9.4 (1.92)	9.4 (1.76)	9.4 (1.93)
% AUDIT ≥ 16^a^	66.7 (448)	67.6 (227)	65.8 (221)	68.2 (229)	65.2 (219)	67.3 (226)	66.1 (222)	66.1 (222)	67.3 (226)	66.7 (224)	66.7 (224)
AUDIT-C score (SD)	9.4 (1.85)	9.4 (1.77)	9.4 (1.92)	9.4 (1.95)	9.4 (1.74)	9.4 (1.86)	9.4 (1.84)	9.4 (1.78)	9.4 (1.92)	9.4 (1.76)	9.4 (1.93)

SD = Standard deviation. PWC = Weekly consumption. Enh. = Enhanced version of module. Min = Minimal version of Module. *Indicates cases where there was a significant difference in a participant characteristic between versions of an intervention module. ^a^Indicates harmful drinkers and drinkers at-risk of alcohol dependence as defined by the AUDIT.

Participants’ characteristics by intervention module are reported in Table 1. In general, characteristics were similar for the enhanced and minimal versions of each intervention module. There were three small but significant differences: users receiving minimal *Normative Feedback* were older (F = 4.23, p = 0.04), and those receiving minimal *Self-monitoring and Feedback* ((**χ**^**2**^ = 4.59, p = 0.04) and *Action Planning* (**χ**^**2**^ = 6.72, p = 0.01) were more likely to be employed.

### Outcomes

#### Primary outcome measure: change in weekly alcohol consumption

Compared with the minimal intensity versions, there were numerically larger decreases in alcohol consumption for enhanced *Normative Feedback*, *Cognitive Bias Re-training* and *Self-monitoring and Feedback*, but there were no significant main effects of intervention module (Table 2).

**Table 2:** Primary outcome: main effects of intervention modules on change in alcohol consumption.

	Mean change in alcohol consumption - Units (SD)				Bayes Factors	
	Enhanced	Minimal	F	P	5 Units	3 Units
Normative Feedback	−4.10 (14.93)	−3.52 (12.87)	0.3	0.59	0.34	0.54
Cognitive Bias Re-training	−4.15 (13.92)	−3.47 (13.95)	0.39	0.53	0.37	0.58
Self-monitoring and Feedback	−4.28 (13.37)	−3.33 (14.48)	0.78	0.38	0.49	0.76
Action Planning	−3.61 (12.22)	−4.01 (15.48)	0.13	0.71	0.16	0.26
Identity Change	−3.02 (13.13)	−4.60 (14.66)	2.16	0.14	0.09	0.15

There was a significant two-way interaction between *Normative Feedback* and *Cognitive Bias Re-training* on weekly alcohol consumption (F = 4.68, p = 0.03, Supplementary Table 3). This indicated that enhanced *Normative Feedback* led to a significant reduction in weekly alcohol consumption only when combined with enhanced *Cognitive Bias Re-training*.

Sensitivity analyses for weekly consumption amongst responders-only when adjusting for app usage and user characteristics showed a similar pattern of results (Supplementary Table 4).

Bayes Factors (BF) showed that the data were insensitive to distinguish an effect for *Normative Feedback* (BF = 0.34), *Cognitive Bias Re-training* (BF = 0.37), *Self-monitoring and Feedback* (BF = 0.49) and *Action Planning* (BF = 0.16) (Table 2). For *Identity Change*, there was strong evidence for the null hypothesis of no effect between versions of the intervention module on change in weekly alcohol consumption (BF = 0.09). A sensitivity analysis with Bayes factors using a smaller expected effect size of a difference of 3 units showed the same pattern of results (Table 2).

#### Secondary outcome measure: Change in full AUDIT score

There were numerically larger decreases in AUDIT scores for enhanced *Normative Feedback*, *Cognitive Bias Re-training*, *Self-monitoring and Feedback* and *Action Planning* but no significant main effects (Table 3). There was a significant two-way interactive effect between *Self-monitoring and Feedback* and *Action Planning* on change in AUDIT score (F = 5.82, p = 0.02, Supplementary Table 5) with the maximum effect occurring when both intervention modules were in their enhanced versions.

**Table 3:** Secondary outcomes: main effects of intervention modules on change in AUDIT score.

	Mean change in AUDIT score (SD)				Bayes factor
	Enhanced	Minimal	F	P	
Normative Feedback	−0.87 (2.68)	−0.61 (2.54)	1.60	0.21	0.54
Cognitive Bias Re-training	−0.77 (2.37)	−0.71 (2.84)	0.10	0.75	0.18
Self-monitoring and Feedback	−0.80 (2.66)	−0.68 (2.56)	0.35	0.56	0.23
Action Planning	−0.88 (2.71)	−0.61 (2.51)	1.75	0.19	0.59
Identity Change	−0.71 (2.71)	−0.77 (2.51)	0.09	0.77	0.11

Sensitivity analyses for change in AUDIT score amongst responders-only when adjusting for app usage and participant characteristics showed the same pattern of results (Supplementary Table 6).

Bayes factors calculated for the main effects of intervention modules on change in AUDIT score (Table 3) indicated that *Cognitive Bias Re-training* (BF = 0.18), *Identity Change* (BF = 0.11) and *Self-monitoring and Feedback* (BF = 0.23) resulted in moderate to anecdotal evidence for the null hypothesis^27^. Bayes factors for *Normative Feedback* (BF = 0.54) and *Action Planning* (BF = 0.59) intervention modules indicated that the data were insensitive to detect this effect.

#### Secondary outcome measure: usage data

Participants used the app for a mean of 11.7 sessions (SD = 13.73), a mean session lasted 4:23 minutes (SD = 4:19). Participants used the app on a mean of eight different days (SD = 8.11) across a mean period of 11 days (SD = 10.92).

A between-subjects ANOVA assessed main and interactive effects of intervention module on app usage (main effects reported in Table 4, interactive effects in Supplementary Table 7). There was a significant main effect of enhanced *Self-monitoring and Feedback* on mean number of sessions (F = 12.73, p < 0.001), but there were no other main effects of intervention module version or two-way interactions on number of sessions. There were no main or interactive effects between intervention module versions on the length of time per session.

**Table 4:** Secondary outcomes: main effects of intervention modules on usage.

	Mean number of sessions (SD)				Mean length of session (SD)			
	Enh	Min	F	P	Enh.	Min	F	P
Normative Feedback	12.33 (14.40)	10.98 (13.02)	1.64	0.2	4:34 (4:39)	4:11 (3:58)	1.36	0.24
Cognitive Bias Re-training	11.77 (14.36)	11.54 (13.10)	0.05	0.82	4:21 (4:21)	4:25 (4:19)	0.04	0.83
Self-monitoring and Feedback	13.53 (15.33)	9.78 (11.66)	12.73	<0.001	4:23 (4:34)	4:23 (4:05)	<0.001	0.99
Action Planning	11.44 (12.79)	11.87 (14.63)	0.17	0.68	4:36 (4:33)	4:09 (4:05)	1.79	0.18
Identity Change	12.14 (13.71)	11.17 (13.76)	0.85	0.36	4:36 (4:41)	4:10 (3:56)	1.74	0.19

Enh = Enhanced version of module, Min = Minimal version of module.

Sensitivity analyses adjusting for number of sessions when assessing length of time per session and for participant characteristics showed the same pattern of results.

#### Secondary outcome measures: usability ratings

Enhanced *Self-monitoring and Feedback* had significantly higher ratings for ‘helpfulness’ (F = 4.39, p = 0.04), ‘recommendation’ (F = 5.02, p = 0.03) and ‘satisfaction’ (F = 6.608, p = 0.01) (Table 5 for main effects of intervention module on usability ratings and Supplementary Table 8 for interactive effects). There were no significant main effects of *Normative Feedback*, *Cognitive Bias Re-training*, *Identity Change* or *Action Planning* on usability ratings.

**Table 5:** Secondary outcomes: main effects of intervention modules on app usability.

	Helpfulness				Ease of use				Recommend to a friend				Satisfaction			
	Enh	Min	F	P	Enh	Min	F	P	Enh	Min	F	P	Enh	Min	F	P
Normative Feedback Mean, (SD)	3.05 (0.88) n = 101	3.04 (1.05) n = 81	0.01	0.9	3.45 (0.97) n = 97	3.62 (1.06) n = 81	0.72	0.4	2.99 (1.23) n = 97	3.10 (1.22) n = 81	0.28	0.6	3.22 (0.95) n = 97	3.17 (1.02) n = 81	0.17	0.68
Cognitive Bias Re-training Mean, (SD)	3.02 (0.98) n = 89	3.06 (0.94) n = 93	0.03	0.86	3.45 (0.97) n = 86	3.60 (1.05) n = 92	0.22	0.64	2.91 (1.23) n = 86	3.16 (1.21) n = 92	1.07	0.3	3.20 (0.97) n = 86	3.20 (1.00) n = 92	0.03	0.86
Self-monitoring & Feedback Mean, (SD)	3.18 (0.93) n = 98	2.88 (0.96) n = 84	4.39	0.04	3.59 (1.00) n = 97	3.46 (1.03) n = 81	1.11	0.29	3.25 (1.22) n = 97	2.79 (1.19) n = 81	5.02	0.03	3.36 (1.00) n = 97	3.00 (0.92) n = 81	6.6	0.01
Action Planning Mean, (SD)	3.04 (1.02) n = 90	3.04 (0.90) n = 92	0.01	0.93	3.56 (1.07) n = 86	3.50 (0.96) n = 92	0.47	0.49	3.08 (1.23) n = 86	3.00 (1.22) n = 92	0.33	0.57	3.26 (1.00) n = 86	3.14 (0.97) n = 92	1.3	0.26
Identity Change Mean, (SD)	3.09 (0.97) n = 96	2.99 (0.94) n = 86	0.17	0.68	3.57 (1.00) n = 93	3.48 (1.02) n = 85	0.02	0.89	3.15 (1.16) n = 93	2.92 (1.28) n = 85	0.4	0.53	3.25 (0.95) n = 93	3.14 (1.01) n = 85	<0.01	0.95

Enh = Enhanced version of module, Min = Minimal version of module.

Sensitivity analysis adjusting for participant characteristics found a similar pattern of results for all usability ratings. When adjusting for app usage, there was the same pattern of results for ‘ease of use’, ‘recommendation’ and ‘satisfaction’ though no main effect of *Self-monitoring and Feedback* on ‘helpfulness’ (F = 2.14, p = 0.15).

## Discussion

This study evaluated enhanced versus minimal versions of five intervention modules (*Normative Feedback*, *Cognitive Bias Re-training*, *Self-monitoring and Feedback*, *Action Planning* and *Identity Change*) within an alcohol reduction app. There were non-significant, but numerically larger decreases in alcohol consumption and AUDIT score for enhanced versions of *Normative Feedback*, *Cognitive Bias Re-training* and *Self-monitoring and Feedback*. There were significant two-way interactions between *Normative Feedback* and *Cognitive Bias Re-training* on weekly alcohol consumption and between *Self-monitoring and Feedback* and *Action Planning* on AUDIT score. Both interactions were in the direction of the maximum reduction occurring when participants received enhanced versions of both modules. Overall, participants used the app for an average of 11.7 sessions and for a mean 4:23 minutes each session. Participants receiving enhanced version of the *Self-monitoring and Feedback* module used the app significantly more times, and rated the app significantly more positively on helpfulness, likelihood to recommend, and satisfaction.

As no main effect of the intervention modules were found, the significant two-way interactions must be interpreted with caution. These particular interactions were not specified a priori, were part of a large number of interaction effects tested, and the interactions are not consistent across closely related outcomes^28^. The inconsistency across the two alcohol-related outcome measures may be due to the different foci of each measure: the primary outcome measure focuses purely on consumption of alcohol, whilst the full AUDIT also accounts for alcohol-related harms and risk of dependency. Alternatively, the inconsistency may be an artefact of modest effects not being reliably detectable across different hypothesis tests. If the inconsistent findings on different outcomes were replicated, then the issue would warrant further examination and theoretical elaboration for why it should be the case. The two-way interactions have not been evaluated in other studies, though a theoretical rationale supports the interactions: the significant two-way interaction between the *Normative Feedback* and *Cognitive Bias Re-training* modules on weekly alcohol consumption is supported by evidence which suggests that interventions targeting both the reflective and automatic motivational systems are more likely to affect behaviour change than either one alone^29,30^; dual-process models of behaviour and the PRIME Theory of Motivation propose that behaviour is determined by motivation and its two systems^31,32^; the *Normative Feedback* module targeted reflective motivation and the *Cognitive Bias Re-training* module targeted automatic motivation. The two-way interaction between *Self-monitoring and Feedback* and *Action Planning* on AUDIT score is also supported by theory and evidence. Control Theory proposes that self-monitoring, feedback and action planning operate synergistically in allowing people to make progress towards goals^33^. Previous findings from alcohol interventions^22^ and a meta-analysis of the effect of self-monitoring on goal attainment^34^ have found the inclusion of more Control Theory congruent BCTs are associated with improved outcomes.

No main effects of the enhanced versions of intervention modules were detected. Accordingly, it was not possible to determine whether the enhanced and minimal module versions were equally helpful or unhelpful. However, as participants in this study were required to complete the AUDIT questionnaire at baseline it may be that the absence of a significant main effect resulted from ‘assessment reactivity’, whereby asking participants about their drinking has been found to reduce subsequent alcohol consumption^35^. Control groups receiving baseline assessment often report reduced consumption at follow-up (e.g.^36^). Students asked to complete the three-item AUDIT-C questionnaire significantly reduced their AUDIT-C score by 0.16 points at follow-up compared with a control group with no assessment^37^. Whilst there is some evidence that assessment (such as the AUDIT-C questionnaire) results in a reduction of alcohol consumption, these reductions are fairly small. The *Drink Less* app had the full AUDIT questionnaire as a standard feature for all users and the intervention modules each had an evidence- and theory-base for reducing excessive alcohol consumption. An effect for these intervention modules was predicted over and above that of assessment reactivity.

In addition to baseline measures of consumption, all participants were prompted to complete their drinking diary each morning in order to increase engagement with all modules of the app. Regular reporting of alcohol consumption has been associated with reduced consumption^38^. Students assigned a set of drinking questionnaires at baseline, 3, 6 and 12 months reduced their AUDIT score and had lower peak blood alcohol content (BAC) levels at follow-up than controls, who were only assigned questionnaires at 12 months^39^.

An unregistered intention-to-treat analysis showed a significant overall reduction in weekly alcohol consumption averaging 3.8 units and a reduction in AUDIT score of 0.7 points (Supplementary Table 9). However, this reduction may be explained by regression to the mean. The motivation to seek out an alcohol reduction app may be greater at times when drinking is particularly high; regression to the mean posits that observations that differ substantially from the true mean tend to be followed by observations closer to the true mean^40^. Regression to the mean may account for some within-participant variation in alcohol consumption over time^41^. However, random allocation to experimental groups means that regression to the mean should affect all groups equally. Therefore, any difference in change between an experimental and control group should be the effect of the experimental group over and above that caused by regression to the mean. The effects of regression to the mean can be minimised by powering the sample size to account for regression to the mean and by using ANCOVA to adjust each follow-up measurement according to their baseline measurement^40^.

### Strengths and limitations

To our knowledge, this is the first trial to examine the effectiveness of an alcohol reduction app on a population of self-directed treatment-seekers. Participants were not recruited for a trial and then given an alcohol reduction app, they sought out an app and were then recruited for a trial. This sample is, therefore, representative of people who wish to reduce their excessive alcohol consumption by way of their own resources and mirrors the real-world situation for most users of behaviour change apps.

The use of a factorial design in the trial allowed multiple simultaneous evaluations to be performed with a relatively small sample. Undertaking these trials consecutively using a traditional RCT would have required considerably more participants and taken considerably more time; findings from which may be have been made obsolete by the rate of technological development^42^. The design of the trial and its analysis allowed each intervention module and its interactions with other modules to be assessed independently. Greater understanding of an intervention’s active ingredients is essential if more effective interventions are to be developed^43^. This study therefore provides an important starting point for building an evidence base about which intervention components are effective for the general population of excessive drinkers.

A limitation of this trial was the high attrition rate, with a follow-up rate of only 27%. Attempts to reduce attrition included having a short follow-up period, emailed reminders, and an in-app option to complete the questionnaire. Longer follow-ups are necessary to detect whether a reduction in consumption has been maintained but a 28-day follow-up period was selected for this screening phase following recommendations on efficiency in the multiphase optimisation strategy^25^. Future research is needed to conduct a definitive randomised control trial with long-term outcomes for the optimised version of the app against a single control group.

DBCIs often suffer from low follow-up rates, which reduces the ability to accurately evaluate their effectiveness and undermines the credibility and validity of inferences from trial findings^44^. Missing data were addressed with an intention-to-treat analysis, which provided a conservative estimate of intervention effectiveness^45^. Better ways of increasing follow-up are likely to have increased the credibility and validity of findings; for example, text reminders have been found to increase response rates for a DBCI by over 13%^46^ and a Cochrane review found financial incentives for completion significantly increased response rates for electronic questionnaires (RR: 1.25; 95% CI: 1.14 to 1.38)^47^. Whilst high attrition rates from follow-ups limit the ability to make inferences from trial findings, apps may still have a public health impact providing they achieve sufficient engagement to promote drinking reduction effectively.

The AUDIT questionnaire is a reliable and standardised alcohol-related outcome measure, which has been validated internationally as a screening test and so allows for direct comparisons between studies from different countries [3]. The AUDIT, or AUDIT-C, have been used as the primary outcome measure in the Screening and Intervention Programme for Sensible drinking (SIPS) trial in primary care^48^ and in multiple DBCIs for alcohol reduction (e.g.^49–51^). The AUDIT questionnaire has limitations including that the consumption questions ask about typical rather than specific consumption. Although responsive to change^52^, our primary outcome measure is likely to have been less sensitive to change than the Alcohol Timeline Followback (TLFB) or graduated frequency. However this means the estimate of intervention effectiveness is likely to be conservative and potentially an underestimate of the effect. The brevity of the AUDIT was a crucial criterion for an outcome measure in a digital trial when increased user burden may increase attrition. The use of a self-report measure for alcohol consumption was another limitation, though there are no objective markers of alcohol consumption that could be used in a digital trial. Furthermore, reviewers have generally concluded that self-reported estimates of alcohol consumption show adequate reliability and validity^53^, and our use of a factorial design made differences in self-reporting bias across conditions unlikely. There are other questionnaires to measure alcohol consumption such as the Alcohol Timeline Followback (TLFB)^54^, though the AUDIT measures alcohol consumption, harms and dependence with few questions. The brevity of the AUDIT is a crucial criterion for an outcome measure in a digital trial when increased user burden may increase attrition.

In a further attempt to keep participant burden to a minimum and increase app engagement, measures to assess potentially mediating variables and test theoretical hypotheses were not included. Therefore, we were unable to assess whether the modules change the mediators they targeted without changing alcohol consumption (i.e. the theoretical assumption was not supported) or whether they failed to change the mediators (i.e. the module did not achieve the putative mechanism of action)^55^. For example, there was no ‘testing’ phase in the Cognitive Bias Re-training module. As a result of this, we could not distinguish whether the lack of main effect was due to the module failing to alter existing cognitive biases or that it altered cognitive biases but had no effect on subsequent alcohol consumption.

Another limitation was that a desire to promote engagement amongst participants receiving minimal versions of intervention modules may have made control conditions too active. Most alcohol reduction apps include few BCTs^14^; which suggests that participants in this study who received minimal versions were effectively receiving usual care in the context of digital support. Therefore, estimates of effectiveness are likely to be conservative compared with a more basic control group, such as one where participants did not have access to the app or received usual care.

### Future Research and Implications

A key aim of this study was to screen intervention components with the aim of informing and optimising the next version of the app. Definitive evidence for the effectiveness of specific intervention components was not found; however, the overall picture indicates that an app retaining the enhanced versions of the *Normative Feedback*, *Cognitive Bias Re-training*, *Self-monitoring and Feedback* and *Action Planning* intervention modules may assist with drinking reduction. A future optimised version of the app would also be informed by a content analysis of user feedback received during the trial, which may also help improve the app’s acceptability and feasibility to users. Future research is needed to conduct a definitive randomised control trial with long-term outcomes for the optimised version of the app against a single control group.

### Data collection recommendations

It was not possible to use commercial software to collect experimental data, as tools such as Google Analytics are limited in the data they collect and cannot easily distinguish between participants and non-participants. Our method was to write code that sent data from the app to an online database (Nodechef) and then use free software for merging and cleaning data (Pandas) to extract the data required. In addition to usage data and follow-up measures, this method enabled the collection of user-entered data, such as the type and quantity of drinks consumed, goals set, and action plans recorded. These data were collected for potential future analysis rather than analysis in this study.

When using custom written data collection software it is strongly recommended that a thorough verification process be undertaken before commencing the trial. It is important to ensure that the randomisation procedure works as expected, that the follow-up measures can be completed and that the data can be extracted from the online database without error. It is also strongly recommended that a comprehensive series of user testing be undertaken in order that the data-entry process is as easy as possible for users. Our user testing was performed internally with members of a UCL research group and externally with a formal usability study amongst 24 real-world users of the app^56^. Findings from the testing process identified a number of issues, which if not resolved may have impeded use of the app and the quality of data collected.

## Conclusions

A version of the *Drink Less* app that includes the *Normative Feedback*, *Cognitive Bias Re-training*, *Self-monitoring and Feedback*, and *Action Planning* intervention modules may assist with drinking reduction though the interactive effects should be interpreted with caution. The app merits further optimisation, retaining these modules, and evaluation in a full trial against a minimal control with long-term outcomes.

## Methods

### Design

A 2 × 2 × 2 × 2 × 2 between-subject full factorial RCT was conducted to evaluate the effectiveness of five intervention modules. The five factors were: 1) Normative Feedback vs minimal version, 2) Cognitive Bias Re-training vs minimal version, 3) Self-monitoring and Feedback vs minimal version, 4) Action Planning vs minimal version, and 5) Identity Change vs minimal version. Randomisation was to one of the (2 × 2 × 2 × 2 × 2 = ) 32 experimental conditions in a block randomisation method. The trial was pre-registered on 13th February 2016: http://www.isrctn.com/ISRCTN40104069.

### Intervention

*Drink Less* is an app designed to support an individual making a serious attempt to reduce their alcohol consumption. The app was made freely available on the UK version of the Apple App Store for all smartphones and tablets running iOS8 or above (app version 1.0.7). The content of the app did not change during the trial.

One core module, *Goal Setting*, was included for all participants as there was a pragmatic, methodological need to structure the app around an activity that would engage users and allow experimental manipulation of other supporting modules. Therefore, the app suggests users set at least one goal to reduce their alcohol consumption and offers access to five intervention modules – *Normative Feedback*, *Cognitive Bias Re-training*, *Self-monitoring and Feedback*, *Action Planning*, and *Identity Change* – to help them achieve their drinking reduction goals.

*Normative Feedback* provided participants with personalised information about how their drinking compared with other people of their age group and gender in the UK. *Cognitive Bias Re-training* aimed to re-train approach biases toward alcohol by way of an approach-avoidance game. *Self-monitoring and Feedback* allowed participants to record their alcohol consumption and provided feedback on their consumption and the consequences of consumption (calories consumed, money spent and effect on mood, productivity and sleep), as well as progress against goals. *Action Planning* allowed participants to set implementation intentions (if-then plans for action that are automatically brought to mind whenever a specified situation is encountered^56^) to reduce their drinking. *Identity Change* helped participants to foster a change in their identity so that they didn’t see being a drinker as a key part of their identity.

The minimal versions (i.e. control condition) varied by module. Participants in the minimal *Normative Feedback* module received brief advice in plain text (from the Public Health England website), as this is the usual control in similar normative feedback interventions. Participants in the minimal *Cognitive Bias Re-training* module received the game, instructions and graph of previous scores though the contingencies differed, whereby both the ‘avoid’ and ‘approach’ trials had 1:1 alcohol and non-alcohol images. Participants in the minimal *Self-monitoring and Feedback* module were able to record their consumption as without this ability they were considered unlikely to use other modules of the app, but they were unable to record the consequences of consumption, nor were they given feedback on consumption or the consequences of consumption. Participants in the minimal *Action Planning* module were able to access a screen of text about action planning but were not able to create action plans on the app, as the aim was to determine whether the features included in the Action Planning module made the module more effective. Participants in the minimal *Identity Change* module received a screen of plain text describing the role of identity in behaviour change and maintenance, though were not helped to foster an identity change.

The navigational structure of the app, as well as details of and the content for these five intervention modules is summarised in Supplementary File 2. A detailed description of all elements of the app is reported in two PhD theses^57,58^.

### Sample and recruitment

Informed consent to participate in the trial was obtained from all participants. Participants were included in analysis if they: had an AUDIT score of 8 or above (indicative of excessive alcohol consumption warranting intervention^59^), confirmed they were making an attempt to reduce their drinking, were 18 or over; lived in the United Kingdom and provided an email address. Participants were excluded from the trial, though could still access the app, if their AUDIT score was less than 8, as this was indicative of low-risk drinking. Excessive drinkers are likely to differ from low-risk drinkers in their response to and needs from an alcohol reduction app; so the decision was taken to focus on excessive drinking as this is the public health priority. People who downloaded the app more than once were removed, with the first case of download retained for the trial.

The study recruited 672 participants to have more than 80% power (alpha 5%, 1:1 allocation, and a two-tailed test) to detect a mean change in alcohol consumption of 5 units between the enhanced and minimal conditions for the main and interactive effects of the five intervention modules^60^. This assumed a mean consumption of 22 units weekly at follow-up in the intervention group, a mean of 27 units in the control group and a SD of 23 units for both (d = 0.22). The sample size was rounded up to the nearest multiple of 32 to ensure even allocation to groups. The estimated effect size was the target as it is comparable with a face-to-face brief intervention^2^, though may be considered unrealistic for a module within a digital intervention. To address the possibility of non-significant results, Bayes factors were calculated to establish the relative likelihood of the null versus the experimental hypothesis given the data obtained.

The app was listed in the iTunes Store and the listing was optimised according to best practices for app store optimisation (e.g. careful selection of keywords, a well-written description and illustrative screenshots^61,62^). The app was promoted through organisations such as Public Health England, Cancer Research UK, online communities of people in the UK wanting to reduce their consumption of alcohol and a link on a popular smoking cessation app (Smoke Free). A prize of £100 was offered in return for entering an email address, in an attempt to decrease the proportion of users who might leave this field blank.

A slow initial pace of recruitment was addressed by increasing the prize offered to users who completed the email field to £500 and placing adverts on Facebook and Google. Recruitment for the trial continued until 672 eligible users (21 per experimental condition) were obtained, after excluding duplicate sign-ups.

### Measures

Baseline measures were the AUDIT questionnaire and a socio-demographic assessment (age, gender, ethnic group, level of education, employment status and current smoking status).

The primary outcome measure was self-reported change in weekly alcohol consumption, calculated as the difference between one-month follow-up and baseline. Weekly alcohol consumption was calculated using a method reported in a previous study^60^. This method recodes the AUDIT-C Q1 (How often do you have a drink containing alcohol?) into number of drinking days per week and the AUDIT-C Q2 (How many drinks do you have on a typical day when you are drinking?) into the average number of units of alcohol consumed on a typical drinking day. These two variables were multiplied to arrive at a total number of units for weekly consumption.

Secondary outcome measures were: self-reported change in full AUDIT score; app usage data, and self-reported app usability measures. Measures of app usage were: number of sessions per user and length of time per session. A user session was defined as a period of app use where the length of inactivity between viewing screens lasted less than 30 minutes (with no minimum or maximum number of screens viewed). For example, if a user stopped using the app at 1 pm and started using it again at 1:29 pm that would count as the same session of use; however, if they started using the app again at 1:30 pm that would count as a new session. This is the approach adopted by popular usage data software, such as Google Analytics^63^. Usability measures collected were: helpfulness, ease of use, satisfaction and likelihood of recommendation to a friend; all assessed using a five point Likert-type scale (‘extremely’, ‘very’, ‘somewhat’, ‘slightly’, ‘not at all’).

### Procedure

Each user was provided with a participant information sheet and asked to consent to participate in the trial on first opening the app. Users who consented to participate were asked to complete the AUDIT and a socio-demographic questionnaire, indicate their reason for using the app (‘interested in drinking less alcohol’ or ‘just browsing’), and provide their e-mail address for follow-up. Users were then given their AUDIT score and informed of their ‘AUDIT risk zone’. At this point, users who met inclusion criteria were randomised to one of 32 experimental conditions in a block randomisation method by an automated algorithm within the app. Users not meeting inclusion criteria were allocated to a separate, non-experimental, condition which provided the enhanced version of each intervention module for ethical purposes and to increase the chance of positive ratings on the app store. Participants were blinded to group allocation. The research team could see group allocation in order to verify the randomisation procedure, but had no contact with participants other than responding to emailed requests for support.

The follow-up questionnaire was sent to participants 28 days after downloading the app and consisted of the AUDIT and usability measures. A 28-day follow-up period was considered sufficient to determine whether enhanced versions of modules were more effective than minimal versions. Participants were sent a maximum of four reminders. Follow-up was undertaken by means of a questionnaire in an online survey tool (Qualtrics) that was emailed to participants, participants could also complete the questionnaire within the app. As both methods of follow-up were private, anonymous and conducted via digital technology, there were no differences that would be likely to affect the participant’s willingness and/or ability to provide accurate information^53^. Duplicate entries were identified through the user’s unique ID, with the earliest complete record used.

### Analysis

The analysis plan was pre-registered on 13^th^ February 2016 (ISRCTN40104069^21^). Socio-demographic and drinking characteristics of participants were reported descriptively. Differences between participant characteristics by intervention module were examined with one-way ANOVAs for continuous variables and 2-sided chi-squared tests for categorical variables.

Main and interactive effects of the five intervention modules on the primary and secondary outcomes were examined with a factorial between-subjects ANOVA. ANCOVAs were conducted in a sensitivity analysis to adjust for chance imbalances in drinking (AUDIT and AUDIT-C score) and socio-demographic characteristics (gender, age, ethnicity, level of education, and employment status).

An intention-to-treat analysis was used for the change in weekly alcohol consumption and change in AUDIT score, such that those lost to follow-up (non-responders) were assumed to be drinking at baseline levels. An intention-to-treat analysis is often used in the evaluation of DBCIs (e.g.^64,65^) to ensure that effect sizes are not over-estimated, as participants who respond well to an intervention may be more likely to respond to follow-up. Sensitivity analyses were conducted among those who completed the follow-up questionnaire (responders) to examine the robustness of the results to assumptions made in the primary analysis. The analysis plan specified imputing missing data from baseline characteristics, though this procedure was not completed as response rates were too low for the method to be valid.

Analysis of the usability ratings involved complete cases. A sensitivity analysis of the usage measure – time per session – was conducted with number of sessions as a covariate to address the potential bias introduced by participants using the app only once (as first time use, which included registration, is likely to be longer than subsequent uses).

Bayes factors were calculated in the event of a non-significant main effect of an intervention module to establish the relative likelihood of the experimental versus the null hypothesis given the data obtained^66^. The use of Bayes factors when analysing data from randomised trials in addition to traditional frequentist statistics provides information about whether the data are insensitive to detect an effect or support the null hypothesis^67^. These can lead to more precise conclusions for non-significant results than are typically obtained using only traditional null hypothesis testing^67^. The Bayes factors are less familiar to many than traditional frequentist statistics and so we pre-planned to use them only when they are most helpful (i.e., in the event of a non-significant result).

Bayes factors were calculated using the online calculator: http://www.lifesci.sussex.ac.uk/home/Zoltan_Dienes/inference/Bayes.htm. The alternative hypotheses were conservatively represented in each case by a half-normal distribution. The standard deviation of a distribution can be specified as an expected effect size, which means, in the case of a half-normal distribution, smaller values are more likely and plausible values have been effectively represented between zero and twice the effect size. The expected effect size for the primary calculation of Bayes factors will reflect that of the power calculation, a reduction of 5 units per week (d = 0.22). For screening purposes to inform retention of the module in future versions of the app, Bayes factors were also calculated for a smaller effect to permit a relative judgment, reflecting a reduction of 3 units per week (d = 0.13).

### Ethical approval

The experimental protocols were approved by the UCL Ethics Committee under the ‘optimisation and implementation of interventions to change health-related behaviours’ project (CEHP/2013/508). All methods were performed in accordance with the guidelines and regulations specified by UCL.

### Availability of data and material

The anonymised dataset is available on the Open Science Framework (https://osf.io/q8mua/) and the app source code is available on request.

### Trial registration

ISRCTN40104069. Registered on 13^th^ February 2016. Available from: http://www.isrctn.com/ISRCTN40104069

## Electronic supplementary material

Supplementary information

## References

[1] Public Health England. *Alcohol treatment in England* 2012–13 (2013).

[2] Kaner, E. F. S. *et al*. Effectiveness of brief alcohol interventions in primary care populations. *Cochrane database Syst*. *Rev*. 10.1002/14651858.CD004148.pub3 (2007). 10.1002/14651858.CD004148.pub3 17443541

[3] Purshouse RC (2013). Modelling the cost-effectiveness of alcohol screening and brief interventions in primary care in England. Alcohol Alcohol.

[4] Angus C, Latimer N, Preston L, Li J, Purshouse R (2014). What are the implications for policy makers? A systematic review of the cost-effectiveness of screening and brief interventions for alcohol misuse in primary care. Front. Psychiatry.

[5] Brown J (2016). Comparison of brief interventions in primary care on smoking and excessive alcohol consumption: a population survey in England. Br. J. Gen. Pract..

[6] Denny CH, Serdula MK, Holtzman D, Nelson DE (2003). Physician advice about smoking and drinking: Are U.S. adults being informed?. Am. J. Prev. Med..

[7] Kaner, E. Brief alcohol intervention: time for translational research. *Addictio*n **105**, 960-1-5 (2010). 10.1111/j.1360-0443.2009.02848.x 20659054

[8] Heather N, Dallolio E, Hutchings D, Kaner E, White M (2004). Implementing routine screening and brief alcohol intervention in primary health care: A Delphi survey of expert opinion. J. Subst. Use.

[9] Taylor CB, Luce KH (2003). Computer- and internet-based psychotherapy interventions. Curr. Dir. Psychol. Sci..

[10] Heather N (2011). Effectiveness of E-self-help interventions for curbing adult problem drinking: a meta-analysis. J. Med. Internet Res..

[11] Khadjesari Z, Murray E, Hewitt C, Hartley S, Godfrey C (2011). Can stand-alone computer-based interventions reduce alcohol consumption? A systematic review. Addiction.

[12] Carey KB, Scott-Sheldon LaJ, Elliott JC, Bolles JR, Carey MP (2009). Computer-delivered interventions to reduce college student drinking: a meta-analysis. Addiction.

[13] Ef, K. *et al*. Personalised digital interventions for reducing hazardous and harmful alcohol consumption in community-dwelling populations | Cochrane. (2015). 10.1002/14651858.CD011479.pub2 PMC6483779 28944453

[14] Crane D, Garnett C, Brown J, West R, Michie S (2015). Behavior Change Techniques in Popular Alcohol Reduction Apps. J. Med. Internet Res..

[15] Liu F (2015). Mobile Phone Intervention and Weight Loss Among Overweight and Obese Adults: A Meta-Analysis of Randomized Controlled Trials. Am. J. Epidemiol..

[16] Lyzwinski LN (2014). A systematic review and meta-analysis of mobile devices and weight loss with an Intervention Content Analysis. Journal of Personalized Medicine.

[17] Gonzalez VM, Dulin PL (2015). Comparison of a smartphone app for alcohol use disorders with an Internet-based intervention plus bibliotherapy: A pilot study. J. Consult. Clin. Psychol..

[18] Hasin DS, Aharonovich E, Greenstein E (2014). HealthCall for the smartphone: technology enhancement of brief intervention in HIV alcohol dependent patients. Addict. Sci. Clin. Pract..

[19] Gustafson DH (2014). A Smartphone Application to Support Recovery From Alcoholism. JAMA Psychiatry.

[20] Gajecki M, Berman AH, Sinadinovic K, Rosendahl I, Andersson C (2014). Mobile phone brief intervention applications for risky alcohol use among university students: A randomized controlled study. Addict. Sci. Clin. Pract..

[21] Garnett C, Crane D, Michie S, West R, Brown J (2016). Evaluating the effectiveness of a smartphone app to reduce excessive alcohol consumption: Protocol for a randomised control trial. BioMed Cent..

[22] Michie S (2012). Identification of behaviour change techniques to reduce excessive alcohol consumption. Addiction.

[23] Garnett C, Crane D, West R, Brown J, Michie S (2015). Identification of Behavior Change Techniques and Engagement Strategies to Design a Smartphone App to Reduce Alcohol Consumption Using a Formal Consensus Method. JMIR mHealth uHealth.

[24] Collins LM, Kugler KC, Gwadz MV (2016). Optimization of Multicomponent Behavioral and Biobehavioral Interventions for the Prevention and Treatment of HIV/AIDS. AIDS Behav..

[25] Collins LM, Murphy SA, Strecher V (2007). The multiphase optimization strategy (MOST) and the sequential multiple assignment randomized trial (SMART): new methods for more potent eHealth interventions. Am. J. Prev. Med..

[26] Pham Q, Wiljer D, Cafazzo JA (2016). Beyond the Randomized Controlled Trial: A Review of Alternatives in mHealth Clinical Trial Methods. JMIR mHealth uHealth.

[27] Jeffreys, H. *The Theory of Probability*. (Oxford University Press, 1961).

[28] Sun X, Briel M, Walter SD, Guyatt GH (2010). Is a subgroup effect believable? Updating criteria to evaluate the credibility of subgroup analyses. Br. Med. J..

[29] Hollands, G. J., Marteau, T. M. & Fletcher, P. C. Non-Conscious Processes in Changing Health-Related Behaviour: A Conceptual Analysis and Framework. *Health Psychol*. *Rev*. 1–14, 10.1080/17437199.2015.1138093 (2016). 10.1080/17437199.2015.1138093 PMC5214381 26745243

[30] Marteau TM, Hollands GJ, Fletcher PC (2012). Changing Human Behavior to Prevent Disease: The Importance of Targeting Automatic Processes. Science (80-.)..

[31] Bechara A (2005). Decision making, impulse control and loss of willpower to resist drugs: a neurocognitive perspective. Nat. Neurosci..

[32] West, Robert, Brown, J., West, R. & Brown, J. *Theory of Addiction*. (John Wiley & Sons., 2013).

[33] Carver CS, Scheier MF (1982). Control theory: a useful conceptual framework for personality-social, clinical, and health psychology. Psychol. Bull..

[34] Harkin B (2016). Does monitoring goal progress promote goal attainment? A meta-analysis of the experimental evidence. Psychol. Bull..

[35] McCambridge, J. & Kypri, K. Can simply answering research questions change behaviour? Systematic review and meta analyses of brief alcohol intervention trials. *PLoS One* **6** (2011). 10.1371/journal.pone.0023748 PMC3187747 21998626

[36] Neighbors C (2010). Efficacy of web-based personalized normative feedback: a two-year randomized controlled trial. J. Consult. Clin. Psychol..

[37] McCambridge J (2013). Alcohol assessment and feedback by email for university students: main findings from a randomised controlled trial. Br. J. Psychiatry.

[38] Helzer JE, Badger GJ, Rose GL, Mongeon JA, Searles JS (2002). Decline in alcohol consumption during two years of daily reporting. J. Stud. Alcohol.

[39] Walters ST, Vader AM, Harris TR, Jouriles EN (2009). Reactivity to alcohol assessment measures: An experimental test. Addiction.

[40] Barnett AG, van der Pols JC, Dobson AJ (2005). Regression to the mean: What it is and how to deal with it. Int. J. Epidemiol..

[41] Finney JW (2008). Regression to the mean in substance use disorder treatment research. Addiction.

[42] Patrick K (2016). The Pace of Technologic Change. Am. J. Prev. Med..

[43] Michie S (2013). The behavior change technique taxonomy (v1) of 93 hierarchically clustered techniques: Building an international consensus for the reporting of behavior change interventions. Ann. Behav. Med..

[44] Eysenbach G (2002). Issues in evaluating health websites in an Internet-based randomized controlled trial. J. Med. Internet Res..

[45] White IR, Horton NJ, Carpenter J, Pocock SJ (2011). Strategy for intention to treat analysis in randomised trials with missing outcome data. BMJ.

[46] Naughton, F., Riaz, M. & Sutton, S. Brief report Response Parameters for SMS Text Message Assessments Among Pregnant and General Smokers Participating in SMS Cessation Trials. 1–5, 10.1093/ntr/ntv266 (2015). 10.1093/ntr/ntv266 PMC4826491 26660264

[47] Brueton VC (2014). Strategies to improve retention in randomised trials: a Cochrane systematic review and meta-analysis. BMJ Open.

[48] Kaner E (2013). Effectiveness of screening and brief alcohol intervention in primary care (SIPS trial): pragmatic cluster randomised controlled trial. Bmj.

[49] Butler SF, Chiauzzi E, Bromberg JI, Budman SH, Buono DP (2003). Computer-Assisted Screening and Intervention for Alcohol Problems inPrimary Care. J. Technol. Hum. Serv..

[50] Sinadinovic K, Wennberg P, Johansson M, Berman AH (2014). Targeting Individuals with Problematic Alcohol Use via Web-Based Cognitive-Behavioral Self-Help Modules, Personalized Screening Feedback or Assessment Only: A Randomized Controlled Trial. Eur. Addict. Res..

[51] Cucciare Ma, Weingardt KR, Ghaus S, Boden MT, Frayne SM (2013). A randomized controlled trial of a web-delivered brief alcohol intervention in Veterans Affairs primary care. J. Stud. Alcohol Drugs.

[52] Bradley KA (1998). The AUDIT alcohol consumption questions: reliability, validity, and responsiveness to change in older male primary care patients. Alcohol. Clin. Exp. Res..

[53] Del Boca FK, Darkes J (2003). The validity of self-reports of alcohol consumption: state of the science and challenges for research. Addiction.

[54] Sobell, L. C. & Sobell, M. B. in *Measuring alcohol consumption* 41–72 (Humana Press, 1992).

[55] Sheeran, P., Klein, W. M. P. & Rothman, A. J. Health Behavior Change: Moving from Observation to Intervention. *Annu*. *Rev*. *Psychol*. **68** (2016). 10.1146/annurev-psych-010416-044007 27618942

[56] Gollwitzer PM (1999). Implementation intentions: Strong effects of simple plans. Am. Psychol..

[57] Garnett, C. Development and evaluation of a theory- and evidence-based smartphone app to help reduce excessive alcohol consumption (unpublished doctoral thesis). (University College London, 2016).

[58] Crane, D. Development and evaluation of a smartphone app to reduce excessive alcohol consumption: Self-regulatory factors. (University College London, 2017).

[59] Reinert DF, Allen JP (2007). The alcohol use disorders identification test: An update of research findings. Alcohol. Clin. Exp. Res..

[60] Kunz FM, French MT, Bazargan-Hejazi S, Al KET (2004). Cost-effectiveness analysis of a brief intervention delivered to problem drinkers presenting at an inner-city hospital emergency department. J. Stud. Alcohol.

[61] The Definitive Guide to App Store Optimization (ASO). Available at: http://www.searchenginejournal.com/definitive-guide-app-store-optimization-aso/78719/. (Accessed: 14th January 2016)

[62] The importance of App Store reviews - Cowly Owl. Available at: http://www.cowlyowl.com/blog/app-store-reviews. (Accessed: 14th January 2016)

[63] Google Analytics. How a web session is defined in Analytics. Available at: https://support.google.com/analytics/answer/2731565?hl=en. (Accessed: 7th November 2017).

[64] Brown J (2014). Internet-based intervention for smoking cessation (StopAdvisor) in people with low and high socioeconomic status: a randomised controlled trial. Lancet. Respir. Med..

[65] Schulz DN (2013). Effects of a Web-based tailored intervention to reduce alcohol consumption in adults: randomized controlled trial. J. Med. Internet Res..

[66] Dienes Z (2014). Using Bayes to get the most out of non-significant results. Front. Psychol..

[67] Beard, E., Dienes, Z., Muirhead, C. & West, R. Using Bayes Factors for testing hypotheses about intervention effectiveness in addictions research. *Addiction* 1–18, 10.1111/add.13501 (2016). 10.1111/add.13501 PMC5111611 27347846

