# Publisher Correction: A smartphone app to reduce excessive alcohol consumption: Identifying the effectiveness of intervention components in a factorial randomised control trial

**DOI:** 10.1038/s41598-018-25185-2

**Published:** 2018-04-27

**Authors:** David Crane, Claire Garnett, Susan Michie, Robert West, Jamie Brown

**Affiliations:** 1 Department of Clinical, Educational and Health Psychology, University College London, London, UK; 2 Department of Behavioural Science and Health, University College London, London, UK

Correction to: *Scientific Reports* 10.1038/s41598-018-22420-8, published online 12 March 2018

The original version of this Article contained a typographical error in the Abstract.

“Excessive drinkers (AUDIT > = 8) were recruited to test enhanced versus minimal (reduced functionality) versions of five app modules in a 25 factorial trial.”

now reads:

“Excessive drinkers (AUDIT > = 8) were recruited to test enhanced versus minimal (reduced functionality) versions of five app modules in a 2^5^ factorial trial.”

This Article also contained errors in the Results section under subheading ‘Participants’.

“There were no significant differences in retention rate between versions of the intervention modules. 16th June and 28th August 2016. Fig. 1 shows a flow chart of users from the trial.”

now reads:

“There were no significant differences in retention rate between versions of the intervention modules. Fig. 1 shows a flow chart of users from the trial.”

These errors have now been corrected in the HTML and PDF versions of the Article.

