# Supplementary material for: A smartphone app to reduce excessive alcohol consumption: Identifying the effectiveness of intervention components in a factorial randomised control trial
Source: Sci Rep. 2018 Mar 12;8:4384. doi: 10.1038/s41598-018-22420-8 (PMC5847600; doi:10.1038/s41598-018-22420-8)
Supplement: Supplementary file 1 — Supplementary information [file 41598_2018_22420_MOESM1_ESM.pdf]

A smartphone app to reduce excessive alcohol consumption: Identifying the effectiveness of intervention components in a factorial randomised control trial

Supplementary information

Author details

David Crane PhD[1*] david.crane.13@ucl.ac.uk

Claire Garnett PhD[1,2*] c.garnett@ucl.ac.uk

Susan Michie DPhil[1,] s.michie@ucl.ac.uk

Robert West PhD[3] robert.west@ucl.ac.uk

Jamie Brown PhD[1, 2] jamie.brown@ucl.ac.uk

* joint first authorship

Supplementary Table 1: Details of intervention modules

		Details of module		BCTs	
Module	Objective	Enhanced	Minimal	Enhanced version	Minimal version
Normative feedback	Inform users of the social drinking norm and alert them to any discrepancy with how they believe their drinking compares with normal to how it actually compares with normal.	Questions assessing how users think they compare with others.<br><br>Infographics illustrating how user's drinking actually compares with other adults and others of same gender and age.	Text on risks of drinking too much (from PHE website)	Social comparison<br><br>Provide normative information about others' behaviour and experiences	Information about health consequences<br><br>Information about emotional consequences
Cognitive bias re-training	Use a form of cognitive bias modification to strengthen cognitive control over the automatic biases to approach alcohol that predict alcohol use that exist amongst the users through an engaging game.	Game with all alcohol related pictures associated with "avoid" and all soft drink pictures associated with "approach".<br><br>Additional section of text on why and how this sort of game is believed to work.	Game with 50% of alcohol related pictures associated with "avoid" and 50% associated with "approach". Same for soft drink pictures.	None	None

		Details of module		BCTs	
Module	Objective	Enhanced	Minimal	Enhanced version	Minimal version
Self-monitoring and feedback	Facilitate easy and on-going recording of alcohol consumption; provide feedback on consumption, consequences of consumption and progress toward goals.	Ability to record drinks, graph showing units consumed calories consumed, amount spent on alcohol. Record mood, productivity, clarity, sleep quality, graph illustrating how they differ on mornings after heavy drinking compared to mornings after light/no drinking. Feedback on progress towards goals: cumulatively as the week progresses, on the past week and on all previous weeks.	Ability to record drinks. No other self-monitoring facilitated. No feedback provided	Review behaviour goals, Discrepancy between current behaviour and goal, Feedback on behaviour, Self-monitoring of behaviour, Self-monitoring of outcomes of behaviour, Feedback on outcomes of behaviour, Salience of consequences, Information about emotional consequences, Social reward, Self-reward	Self-monitoring of behaviour
Action planning	Allow users to create implementation intentions for dealing with difficult drinking situations.	Create implementation intentions, review implementation intentions already created, gain understanding of why to set implementation intentions.	Gain understanding of why to set implementation intentions only.	Action planning,<br><br>Credible source	None

		Details of module		BCTs	
Module	Objective	Enhanced	Minimal	Enhanced version	Minimal version
Identity change	Help users foster a change in their identity so that users do not see themselves as "drinkers" as a key part of their identity which should aid their behaviour change attempt.	Memos – record messages about drinking or when drunk too much to watch in the future<br><br>"I am": identifying and considering those values that are important to you, and whether you do not live up to those values when you have drunk too much.<br><br>Flipsides of drinking:<br><br>Providing examples of the negative sides of positive alcohol expectancies.	Text on how identity is an important factor in how we behave and advise to think about the undesired consequences of drinking too much.	Identification of self as role model<br><br>Incompatible beliefs<br><br>Valued self-identity<br><br>Identity associated with changed behaviour<br><br>Information about health/social/emotional consequences<br><br>Salience of consequences<br><br>Anticipated regret<br><br>Pros and cons<br><br>Framing/reframing	None

Supplementary Table 2: Experimental group matrix

Group	Self-monitoring and Feedback	Action Planning	Normative Feedback	Identity Change	Cognitive Bias Re-training
1	Enhanced	Enhanced	Enhanced	Enhanced	Enhanced
2	Enhanced	Minimal	Enhanced	Enhanced	Enhanced
3	Minimal	Enhanced	Enhanced	Enhanced	Enhanced
4	Minimal	Minimal	Enhanced	Enhanced	Enhanced
5	Enhanced	Enhanced	Enhanced	Enhanced	Minimal
6	Enhanced	Minimal	Enhanced	Enhanced	Minimal
7	Minimal	Enhanced	Enhanced	Enhanced	Minimal
8	Minimal	Minimal	Enhanced	Enhanced	Minimal
9	Enhanced	Enhanced	Enhanced	Minimal	Enhanced
10	Enhanced	Minimal	Enhanced	Minimal	Enhanced
11	Minimal	Enhanced	Enhanced	Minimal	Enhanced
12	Minimal	Minimal	Enhanced	Minimal	Enhanced
13	Enhanced	Enhanced	Enhanced	Minimal	Minimal
14	Enhanced	Minimal	Enhanced	Minimal	Minimal
15	Minimal	Enhanced	Enhanced	Minimal	Minimal
16	Minimal	Minimal	Enhanced	Minimal	Minimal
17	Enhanced	Enhanced	Minimal	Enhanced	Enhanced
18	Enhanced	Minimal	Minimal	Enhanced	Enhanced
19	Minimal	Enhanced	Minimal	Enhanced	Enhanced
20	Minimal	Minimal	Minimal	Enhanced	Enhanced
21	Enhanced	Enhanced	Minimal	Enhanced	Minimal
22	Enhanced	Minimal	Minimal	Enhanced	Minimal
23	Minimal	Enhanced	Minimal	Enhanced	Minimal
24	Minimal	Minimal	Minimal	Enhanced	Minimal
25	Enhanced	Enhanced	Minimal	Minimal	Enhanced
26	Enhanced	Minimal	Minimal	Minimal	Enhanced
27	Minimal	Enhanced	Minimal	Minimal	Enhanced
28	Minimal	Minimal	Minimal	Minimal	Enhanced
29	Enhanced	Enhanced	Minimal	Minimal	Minimal
30	Enhanced	Minimal	Minimal	Minimal	Minimal
31	Minimal	Enhanced	Minimal	Minimal	Minimal
32	Minimal	Minimal	Minimal	Minimal	Minimal

E = Enhanced; M = minimal

Supplementary Table 3: Full results of factorial ANOVA on primary outcome measure

	F	P
Normative Feedback (NF)	0.30	0.59
Cognitive Bias Re-training (CBR)	0.39	0.53
Identity Change (IC)	2.16	0.14
Self-monitoring and Feedback (SM)	0.78	0.38
Action Planning (AP)	0.14	0.71
NF x CBR	4.68	0.03
NF x IC	0.81	0.37
NF x SM	2.20	0.14
NF x AP	0.08	0.78
CBR x IC	0.94	0.33
CBR x SM	2.80	0.10
CBR x AP	0.01	0.93
IC x SM	0.39	0.54
IC x AP	0.04	0.84
SM x AP	0.22	0.64
NF x CBR x IC	1.86	0.17
NF x CBR x SM	0.03	0.87
NF x CBR x AP	4.94	0.03
NF x IC x SM	0.03	0.87
NF x IC x AP	0.29	0.59
NF x SM x AP	0.14	0.71
CBR x IC x SM	0.00	0.96
CBR x IC x AP	0.28	0.60
CBR x SM x AP	2.68	0.10
IC x SM x AP	0.52	0.47
NF x CBR x IC x SM	1.08	0.30
NF x CBR x IC x AP	0.01	0.93
NF x CBR x SM x AP	0.49	0.48
NF x IC x SM x AP	0.05	0.82
CBR x IC x SM x AP	0.85	0.36
NF x CBR x IC x SM x AP	0.12	0.73

Supplementary Table 4: Sensitivity analysis on primary outcome – main effects of intervention modules on change in past week alcohol consumption among responders-only

	Mean change in past week alcohol consumption, units per week (SD)			
	Intensive	Minimal	F	P
Normative Feedback	-14.1 (25.07)	-14.6 (23.03)	0.12	0.73
Cognitive Bias Re-training	-16.0 (23.73)	-12.7 (24.47)	0.13	0.72
Action Planning	-13.9 (20.87)	-14.6 (26.92)	0.02	0.90
Self-monitoring & Feedback	-14.8 (21.57)	-13.7 (26.92)	0.05	0.82
Identity Change	-10.8 (23.17)	-18.2 (24.65)	4.26	0.04

Supplementary Table 5: Full results of factorial ANOVA on secondary outcomes– change in AUDIT score

	AUDIT score	
	F	P
Normative Feedback (NF)	1.60	0.21
Cognitive Bias Re-training (CBR)	0.11	0.75
Identity Change (IC)	0.09	0.77
Self-monitoring and Feedback (SM)	0.35	0.56
Action Planning (AP)	1.75	0.19
NF x CBR	1.46	0.23
NF x IC	0.22	0.64
NF x SM	0.78	0.38
NF x AP	1.19	0.28
CBR x IC	0.28	0.60
CBR x SM	0.50	0.48
CBR x AP	1.60	0.21
IC x SM	0.46	0.50
IC x AP	2.34	0.13
SM x AP	5.82	0.02
NF x CBR x IC	0.01	0.91
NF x CBR x SM	0.03	0.86
NF x CBR x AP	0.07	0.79
NF x IC x SM	0.20	0.66
NF x IC x AP	0.03	0.86
NF x SM x AP	1.39	0.24
CBR x IC x SM	0.02	0.88
CBR x IC x AP	0.09	0.77
CBR x SM x AP	0.35	0.56
IC x SM x AP	0.54	0.46
NF x CBR x IC x SM	1.06	0.30
NF x CBR x IC x AP	1.99	0.16
NF x CBR x SM x AP	0.03	0.86
NF x IC x SM x AP	0.20	0.66
CBR x IC x SM x AP	0.54	0.46
NF x CBR x IC x SM x AP	0.31	0.58

Supplementary Table 6: Sensitivity analysis on secondary outcome – main effects of intervention modules on change in AUDIT score among responders-only

	Mean change in AUDIT score (SD)			
	Intensive	Minimal	F	P
Normative Feedback	-3.1 (4.34)	-2.7 (4.77)	0.69	0.41
Cognitive Bias Re-training	-3.2 (3.94)	-2.6 (5.01)	0.28	0.60
Action Planning	-3.5 (4.51)	-2.3 (4.49)	2.67	0.11
Self-monitoring & Feedback	-2.8 (4.41)	-3.0 (4.70)	0.04	0.84
Identity Change	-2.6 (4.72)	-3.2 (4.31)	1.02	0.32

Supplementary Table 7: Full results table for secondary outcome measure – app usage

	Number of sessions		Length per session	
	F	P	F	P
Normative Feedback (NF)	1.64	0.20	1.64	0.20
Cognitive Bias Re-training (CBR)	0.05	0.82	0.05	0.82
Identity Change (IC)	0.86	0.36	0.86	0.36
Self-monitoring and Feedback (SM)	12.73	<0.001	12.73	<0.001
Action Planning (AP)	0.17	0.68	0.17	0.68
NF x CBR	0.40	0.53	0.40	0.53
NF x IC	2.33	0.13	2.33	0.13
NF x SM	0.92	0.34	0.92	0.34
NF x AP	2.49	0.12	2.49	0.12
CBR x IC	0.14	0.70	0.14	0.70
CBR x SM	0.24	0.62	0.24	0.62
CBR x AP	0.26	0.61	0.26	0.61
IC x SM	0.00	0.96	0.00	0.96
IC x AP	0.19	0.66	0.19	0.66
SM x AP	0.03	0.86	0.03	0.86
NF x CBR x IC	0.86	0.36	0.86	0.36
NF x CBR x SM	<0.001	1.00	<0.001	1.00
NF x CBR x AP	0.12	0.73	0.12	0.73
NF x IC x SM	0.12	0.73	0.12	0.73
NF x IC x AP	0.28	0.60	0.28	0.60
NF x SM x AP	6.37	0.01	6.37	0.01
CBR x IC x SM	0.54	0.46	0.54	0.46
CBR x IC x AP	1.81	0.18	1.81	0.18
CBR x SM x AP	0.60	0.44	0.60	0.44
IC x SM x AP	2.93	0.09	2.93	0.09
NF x CBR x IC x SM	5.73	0.02	5.73	0.02
NF x CBR x IC x AP	1.20	0.27	1.20	0.27
NF x CBR x SM x AP	0.29	0.59	0.29	0.59
NF x IC x SM x AP	0.01	0.91	0.01	0.91
CBR x IC x SM x AP	0.48	0.49	0.48	0.49
NF x CBR x IC x SM x AP	0.24	0.63	0.24	0.63

Supplementary Table 8: Full results table for secondary outcome measure – usability ratings

	Helpfulness N=182		Ease of use N=178		Recommendation N=178		Satisfaction N=178	
	F	P	F	P	F	P	F	P
Normative Feedback (NF)	0.02	0.90	0.72	0.40	0.28	0.60	0.17	0.68
Cognitive Bias Re-training (CBR)	0.03	0.86	0.22	0.64	1.07	0.30	0.03	0.86
Self-monitoring and Feedback (SM)	4.39	0.04	1.11	0.29	5.02	0.03	6.60	0.01
Action Planning (AP)	0.01	0.93	0.47	0.49	0.33	0.57	1.30	0.26
Identity Change (IC)	0.17	0.68	0.02	0.89	0.40	0.53	0.00	0.95
NF x CBR	0.42	0.52	3.81	0.05	0.02	0.88	0.09	0.76
NF x IC	0.13	0.72	0.01	0.91	0.01	0.92	0.02	0.90
NF x SM	0.08	0.79	1.61	0.21	3.11	0.08	1.58	0.21
NF x AP	2.52	0.12	0.49	0.49	0.04	0.85	0.21	0.65
CBR x IC	0.73	0.40	<0.001	1.00	0.08	0.78	0.02	0.89
CBR x SM	0.33	0.57	0.22	0.64	0.00	0.98	0.29	0.59
CBR x AP	0.03	0.87	<0.001	0.99	0.01	0.91	0.05	0.82
IC x SM	<0.001	1.00	0.34	0.56	0.14	0.71	0.00	0.96
IC x AP	0.01	0.93	0.21	0.64	0.01	0.94	0.00	0.96
SM x AP	0.12	0.73	0.63	0.43	0.20	0.65	0.05	0.82
NF x CBR x IC	0.18	0.67	0.68	0.41	0.25	0.62	0.11	0.74
NF x CBR x SM	1.30	0.26	1.12	0.29	0.15	0.70	0.52	0.47
NF x CBR x AP	0.93	0.34	0.51	0.48	0.88	0.35	0.15	0.70
NF x IC x SM	0.66	0.42	2.64	0.11	1.39	0.24	3.10	0.08
NF x IC x AP	1.92	0.17	0.19	0.66	1.13	0.29	0.00	0.95
NF x SM x AP	1.40	0.24	1.59	0.21	<0.001	1.00	0.00	0.98
CBR x IC x SM	0.65	0.42	0.22	0.64	0.07	0.80	0.49	0.49
CBR x IC x AP	0.27	0.61	0.03	0.86	0.05	0.82	0.00	0.97
CBR x SM x AP	1.43	0.23	0.71	0.40	0.09	0.76	0.38	0.54
IC x SM x AP	8.44	0.00	2.50	0.12	3.61	0.06	10.62	0.00
NF x CBR x IC x SM	0.66	0.42	0.17	0.68	0.13	0.72	0.15	0.70
NF x CBR x IC x AP	2.32	0.13	0.43	0.52	3.37	0.07	0.48	0.49
NF x CBR x SM x AP	0.08	0.77	0.03	0.88	0.28	0.60	0.58	0.45
NF x IC x SM x AP	2.64	0.11	0.83	0.37	1.10	0.30	3.99	0.05
CBR x IC x SM x AP	0.63	0.43	0.37	0.54	1.53	0.22	0.95	0.33
NF x CBR x IC x SM x AP	0.30	0.58	0.88	0.35	2.47	0.12	1.07	0.30

Supplementary Table 9: Overall effect on outcome measures

Outcome measure		N	Mean (SD)	t	p
Change in past week alcohol consumption, units per week	ITT	672	-3.8 (13.93)	-7.09	<0.001
	Responders only	179	-14.3 (24.10)	-7.94	<0.001
Change in AUDIT score	ITT	672	-0.7 (2.61)	-7.36	<0.001
	Responders only	172	-2.9 (4.53)	-8.39	<0.001
Helpfulness rating		182	3.0 (0.96)	0.62	0.54
Ease of use rating		178	3.5 (1.01)	6.98	<0.001
Recommendation rating		178	3.0 (1.22)	0.43	0.67
Satisfaction rating		178	3.2 (0.98)	2.68	<0.01

Selecting modules for evaluation

The initial selection of modules for evaluation was based on four main sources of evidence: i) examination of the BCTs used in alcohol interventions [1]; ii) a systematic review of the evidence of the effectiveness of digital technologies for reducing excessive alcohol consumption [2]; iii), a formal consensus-building study with experts in the fields of alcohol or behaviour change to identify the behaviour change techniques thought most likely to be effectiveness as reducing alcohol consumption in an app [3]; and iv), a content analysis of the behaviour change techniques within existing popular alcohol reduction apps [4]. On the basis of this systematic development work, the following five modules were selected as high priority for experimental manipulation evaluation in a factorial design: Normative Feedback; Cognitive Bias Re-training; Self-monitoring and Feedback; Action Planning, and Identity Change. We elaborate the reasons for each selection below. A detailed description of all elements of the app is reported in two PhD theses [5,6].

Normative Feedback

Normative feedback is personalised feedback on how an individual's behaviour compares with the behaviour of others. Providing normative feedback can reduce subsequent alcohol use [7–12], indicating that normative misperceptions (underestimating own alcohol use compared with others) plays a role in excessive alcohol consumption. Research has shown that normative misperceptions exist in the general population [13] as well as in heavy drinkers [8,9] and college/university students [10,11,14–16]. Theoretical evidence for the role of normative misperceptions in excessive alcohol consumption come from Social Norms theory [17]. This theory predicts that people behave in a way that attempts to conform to the perceived norm. This can result in people behaving in ways that are not consistent with their own beliefs and values in their attempt to reach the perceived norm [18]. Providing feedback in relation to people was also identified by alcohol and behaviour change experts as an

intervention component likely to be effective at reducing excessive alcohol consumption in an app [3].

Cognitive Bias Re-training

Dual process theories of addiction [19–21] suggest that excessive alcohol consumption occurs, in part, due to automatic processes when the impulses to drink overcome the inhibitory response not to [22].

These automatic biases in information processing of alcohol-related cues or stimuli have been found to predict alcohol use [23,24], though are largely unaffected by interventions targeting changing conscious information or processes [25,26]. Cognitive bias re-training has been found to be effective at altering these automatic cognitive biases [27–31] and some studies have also found associated impacts on subsequent alcohol use [29,30,32,33]. The intervention strategy chosen for this module is to re-train approach biases, with the aim of changing the tendency to approach alcohol and alcohol-related stimuli to an ‘avoid’ bias. Retraining these approach biases has been shown to have a greater efficacy in reducing alcohol consumption [29–31] than retraining other cognitive biases such as attentional biases [34].

Self-monitoring and Feedback

Self-monitoring and Feedback are both recommended as effective techniques for alcohol reduction by NICE clinical guidance [35]. Self-monitoring has been found an effective BCT in weight management [36,37] and alcohol interventions [1]. Feedback is a key component of face-to-face brief alcohol interventions [38], is commonly included in DBCIs [2] and augments the effect of self-monitoring and feedback [39,40]. Self-monitoring and Feedback were both ranked highly by alcohol and behaviour change experts as behaviour change techniques likely to be effective in an alcohol reduction app [3]. The BCTs are also component elements of Control Theory [41], which posits that self-monitoring and feedback

allow determination to be made of the current position in relation to a goal and the progress toward it. Behaviour change interventions which include self-monitoring in combination with at least one of the other Control Theory congruent BCTs have been found to be significantly more effective than interventions not including those techniques [42–44].

Action Planning

Action planning is recommended as an effective technique by NICE clinical guidance [35]. Action planning is also a component of Control Theory, in that it facilitates the reduction of discrepancies between goals and observed behaviour [41]. Action plans detailing the steps necessary to achieve a goal have been found to increase physical activity [45], enhance behaviour change in patients [46] and reduce alcohol consumption [47–49]. 'Implementation intentions', a form of action plan that enable the setting of if/then conditions for future events [50], increased goal-attainment rates for health behaviour such as regular breast examinations [51], engaging in exercise [52] and alcohol reduction [47–49]. Action planning was also ranked highly by alcohol and behaviour change experts as likely to be effective in an alcohol reduction app [3].

Identity Change

Excessive drinking is central to many peoples' sense of self, particularly students [53], and identity has been proposed as a motivational factor for behaviours by a number of theories [54–56], including the PRIME theory of motivation which proposes that identity is a source of motives, self-regulation and stability of behaviour [57]. Identity (group, social and/or individual) was also identified in a consensus approach as a theoretical domain to explain behaviour change [58]. The relationship between identity and behaviour change has not been investigated in the field of alcohol research, though there is evidence from the smoking cessation literature that identity change (adopting an identity that is incongruent with the undesired behaviour) may be an effective intervention technique. A systematic analysis of

English Stop Smoking Services treatment manuals found that ‘strengthening an ex-smoker identity’ was associated with 4-week abstinence rates (both carbon-monoxide verified and self-reported) [59]. A positive smoker identity was present in a minority of smokers in England and predicted failure to make a smoking quit attempt at six months and so may be an important barrier to behaviour change [60]. A meta-ethnography also found that the nature of a smoker’s identity can play an important role in smoking cessation [61].

Navigational structure of the app, details of intervention modules, full content specification for core features of the Drink Less app, full content specification for intervention modules of the Drink Less and experimental group matrix

Supplementary Figure 1: Navigational structure of the Drink Less app

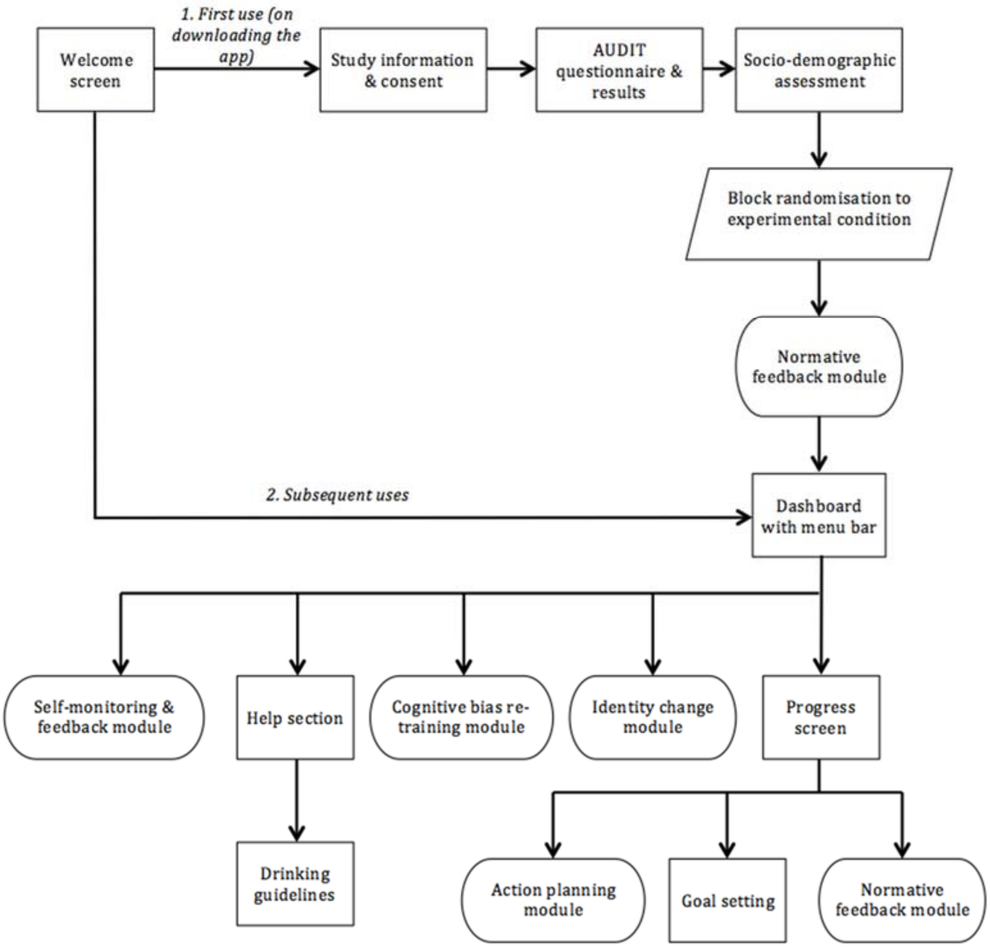

Supplementary Table 1: Details of intervention modules

Intervention module	Objective	Details of module High	Details of module Low	BCTs included in 'high' version	BCTs included in 'low' version
Self-monitoring and feedback	Facilitate easy and on-going recording of alcohol consumption; provide feedback on consumption, consequences of consumption and progress toward goals.	Ability to record drinks, graph showing units consumed calories consumed, amount spent on alcohol. Record mood, productivity, clarity, sleep quality, graph illustrating how they differ on mornings after heavy drinking compared to mornings after light/no drinking. Feedback on progress towards goals: cumulatively as the week progresses, on the past week and on all previous weeks.	Ability to record drinks. No other self-monitoring facilitated. No feedback provided	Review behaviour goals, Discrepancy between current behaviour and goal, Feedback on behaviour, Self-monitoring of behaviour, Self-monitoring of outcomes of behaviour, Feedback on outcomes of behaviour, Salience of consequences, Information about emotional consequences, Social reward, Self-reward	Self-monitoring of behaviour
Action planning	Allow users to create implementation intentions for dealing with difficult drinking situations.	Create implementation intentions, review implementation intentions already created, gain understanding of why to set implementation intentions.	Gain understanding of why to set implementation intentions only.	Action planning,<br><br>Credible source	None

Normative feedback	Inform users of the social drinking norm and alert them to any discrepancy with how they believe their drinking compares with normal to how it actually compares with normal.	Questions assessing how users think they compare with others.<br><br>Infographics illustrating how user's drinking actually compares with other adults and others of same gender and age.	Text on risks of drinking too much (from PHE website)	Social comparison<br><br>Provide normative information about others' behaviour and experiences	Information about health consequence s<br><br>Information about emotional consequence s
Identity change	Help users foster a change in their identity so that users do not see themselves as "drinkers" as a key part of their identity which should aid their behaviour change attempt.	Memos – record messages about drinking or when drunk too much to watch in the future<br><br>"I am": identifying and considering those values that are important to you, and whether you do not live up to those values when you have drunk too much.<br><br>Flipsides of drinking:<br><br>Providing examples of the negative sides of positive alcohol expectancies.	Text on how identity is an important factor in how we behave and advise to think about the undesired consequences of drinking too much.	Identification of self as role model<br><br>Incompatible beliefs<br><br>Valued self-identity<br><br>Identity associated with changed behaviour<br><br>Information about health/social/emotiona l consequences<br><br>Salience of consequences<br><br>Anticipated regret<br><br>Pros and cons<br><br>Framing/reframing	None

Cognitive bias re-training	Use a form of cognitive bias modification to strengthen cognitive control over the automatic biases to approach alcohol that predict alcohol use that exist amongst the users through an engaging game.	Game with all alcohol related pictures associated with “avoid” and all soft drink pictures associated with “approach”.<br><br>Additional section of text on why and how this sort of game is believed to work.	Game with 50% of alcohol related pictures associated with “avoid” and 50% associated with “approach”. Same for soft drink pictures.	None	None

Full content specification for core features of the Drink Less app

Screen	Text
**Information sheet**	Before you decide whether or not to take part in this study it is important for you to understand why the research is being done and what it will involve. Please read the following information and email us if anything is unclear or if you would like more information.<br><br>1. The purpose of the study and eligibility<br>This study aims to evaluate a smartphone application (Drink Less) to help people reduce their alcohol consumption. We want to learn about the effectiveness of different intervention components and users' experiences with using the app. You must be over 18 years old to be eligible for this study.<br>2. What will happen if you decide to take part?<br>The programme will randomly allocate you into a group, and you will receive a number of different intervention components, which will be based on a combination of evidence-based behaviour change techniques. You will be encouraged to use the app daily, to use the different intervention components of the app and to monitor your drinking. If you provide us with your email address, we will email you to ask questions about the app and your drinking 3 months from today.<br>3. Possible risks or discomfort<br>There are no possible risks or discomfort to you for taking part.

4. Possible benefits

   Reducing your drinking levels has a number of health and financial benefits, amongst others.

5. Financial considerations

   There are no financial costs to you for taking part.

6. Confidentiality

   All data from this study will be treated as strictly confidential and handled in accordance with the Data Protection Act 1998. For your protection, we will assign you a unique number that will be used to label all information. Results of this study will be written up for scientific publications, PhD thesis and conference presentations. Results will be available upon request via e-mail (c.garnett.12@ucl.ac.uk or david.crane.13@ucl.ac.uk), and through open access scientific literature. Any records or data obtained as a result of your participation may be checked by the group that oversees research to make sure that human participants are protected.

7. Termination of research study

   You are free to choose whether or not to take part in this study. You can choose to stop participating at any time without giving a reason.

8. Ethics review

   This study has been approved by University College London's Psychology and Language Sciences Departmental Ethics

Committee (Project ID: CEHP/2013/508). Principal Investigator: Professor Susan Michie (s.michie@ucl.ac.uk)

9. Further information and contact details

   If you would like to receive any further information or have any questions, please contact Professor Susan Michie (s.michie@ucl.ac.uk), Claire Garnett (c.garnett.12@ucl.ac.uk) or David Crane (david.crane.13@ucl.ac.uk).

10. Comments or concerns during the study

    If you have any comments or concerns you should discuss these with the Principal Investigator (s.michie@ucl.ac.uk). If you wish to complain further, you should email the Chair of the UCL Committee for the Ethics of Non-NHS Human Research (gradschoolhead@ucl.ac.uk) who will take the complaint forward as necessary.

Consent form

If you do not want to take part in this study, please close the app now. If you would like to take part, please indicate that you consent to do so. By tapping on the consent button below you are indicating that:

- You have read the information pages, had an opportunity to ask questions, and are happy that you understand the study and what it involves for you.
- You realise that you can withdraw from the study at any time.
- You agree that the findings of the study can be published but that you will not be identified.

- All the information you provide will be treated as strictly confidential and handled in accordance with the provisions of the Data Protection Act 1998.

[‘Give consent’ button]

Drinking assessment (AUDIT Questionnaire)

1. Gender

   Male/Female
2. How often do you have a drink containing alcohol?

   Never/Monthly or less/2 to 4 times a month/2 or 3 times a week/4 or more times a week
3. How many units of alcohol do you have on a typical day when you are drinking?

   1-2/3-4/5-6/7-9/10+
4. How often do you have six or more units of alcohol on one occasion?

   Never/Less than monthly/Monthly/Weekly/Daily or almost daily
5. How often during the last year have you found that you were unable to stop drinking once you had started?

   Never/Less than monthly/Monthly/Weekly/Daily or almost daily
6. How often during the last year have you failed to do what was expected from you because of drinking?

   Never/Less than monthly/Monthly/Weekly/Daily or almost daily
7. How often during the last year have you needed a first drink in the morning to get yourself going after a heavy drinking session?

   Never/Less than monthly/Monthly/Weekly/Daily or almost daily

8. How often during the last year have you had a feeling of guilt or remorse after drinking?

   Never/Less than monthly/Monthly/Weekly/Daily or almost daily

9. How often during the last year have you been unable to remember what happened the night before because you had been drinking?

   Never/Less than monthly/Monthly/Weekly/Daily or almost daily

10. Have you or someone else been injured as the result of your drinking?

    No/Yes, but not in the last year/Yes, during the last year

11. Has a relative, friend, or a doctor or other health worker been concerned about your drinking or suggested you cut down?

    No/Yes, but not in the last year/Yes, during the last year

**Feedback on drinking**	**AUDIT Score**	**Feedback given**
	Between 0-7 {Green text)	Your results indicate you're not at risk of physical and/or psychological alcohol-related harm.<br><br>Your score was X which lies in the range of 0-7 for this risk zone.
	Between 8-15 (Yellow text)	Your results indicate you're putting yourself at increasing risk of physical and/or psychological alcohol-related harm.<br><br>Your score was X which lies in the range of 8-15 for this risk zone.
	Between 16-19 (Orange text)	Your results indicate you're likely to be experiencing physical and/or psychological alcohol-related harm.<br><br>Your score was X which lies in the range of 16-19 for this risk zone.
	Between 20-40 (Red text)	You results indicate the possibility of alcohol dependence. You are welcome to continue to use this app though we strongly advise you to contact your GP for further support.

Your score was X which lies in the range of 20-40 for this risk zone.

Create and view goals

Goals main screen

I want to drink less because… [User enters text]

Links to:

[Set and view goals]

[How to set good goals]

Set and view goals

'Active goals'

Lists active goal(s) set by user

Good goals are specific and a little challenging. Not too challenging though, because often the hardest thing about making a change is sticking with it. So it's important to keep your goals realistic. You can alter them at any time if you find they're too difficult or too easy.

Link to:

[Set a new goal]

Information button (i)

This screen lists all your active and any previous goals. If it's empty, start by tapping the green 'Set a new goal' button at the bottom. If you've set a goal, the white circle fills up as the week progresses. Tap this or the text to see your success over the weeks. You can amend

your goal by tapping Edit (in the top right) and then the goal itself. Only tap the red circle if you want to delete it.

Previous goals

List of goal(s) previously set by user and not currently active

Link to:

[Set a new goal]

Information button (i)

Any goals you've deleted are listed here. Tap a goal to see your success over the weeks. Tap Edit and then the red circle to re-activate this goal or delete it entirely.

Set a new goal

Type: Units/ Spending/ Alcohol free days/ Calories

Max: (Defaults were, in the order above: 14/No default/1,100/3)

Recurring: On/ Off (defaults to on)

[Save]

Information button (i)

Type: You can set goal for how many alcohol free days you want each week, the maximum number of units you want to drink, the

maximum amount you want to spend or the maximum number of calories you want to consume. All relate to a week's consumption.

Max: Enter a figure for the type of goal you've set. For example: 3 alcohol free days, 14 units, £20 or 1100 calories.

Recurring: Leave this on and the goal will start again each week.

How to set good goals

Good goals are specific and a little challenging. Not too challenging though, because often the hardest thing about making a change is sticking with it. So it's important to keep your goals realistic. You can alter them at any time if you find they're too difficult or too easy.

You can set goals for:

1. Number of alcohol free days a week
2. The total amount of alcohol you drink
3. The number of calories you get from alcohol
4. The money you spend on alcohol.

We suggest you start with a couple and take it from there.

**About alcohol -> Useful information**	**Drinking guidelines**<br>The government's drinking guidelines have three key recommendations.

1. **Limit your weekly drinking to 14 units**

   Whether you are a man or a woman try not to regularly drink

more than 14 units of alcohol per week. If you drink as much as 14 units per week, it’s best to spread this evenly over 3-5 days but keep 2-4 days alcohol free. See Help > Units guide.

2. **Avoid drinking more than 6 units on any one occasion**

   Limit the amount of alcohol you drink to less than 6 units on any one occasion, if necessary by drinking more slowly, drinking with food or alternating with a soft drink

3. **Do not drink at all if you are pregnant**

   Any amount of alcohol can harm your baby, so it is best not to drink at all.

You can read the full guidelines here [link to uk government drinking guidelines]

Harms of drinking

Harms of drinking

Alcohol increases your chance of experiencing:

Short term effects

- Alcohol poisoning
- Memory loss
- Fatigue
- Depression
- Unsafe sex
- Impotence

- Injury

Long term effects

- Cancer
- Heart disease
- Liver disease
- Stroke
- High blood pressure
- Diabetes
- Reduced fertility
- Insomnia
- Social problems
- Causing or being the victim of violence and antisocial behaviour
- Problems with relationships
- Unplanned time off work or college
- Loss of personal possessions (left your phone in the pub? Again?)

See here for more details [LINK to NHS webpage ‘the risks of drinking too much’]

Benefits of not drinking

Benefits of not drinking

Here are just some of the benefits of not drinking (or drinking less):

- Feel better in the mornings
- Get more and better quality sleep

- Improve your overall mood
- Improve your health
- Save money
- Live longer
- Reduce the likelihood of developing over 60 medical conditions (see *Harms of drinking* for a small sample)

See here for more details [link to NHS webpage ‘tips on cutting down’]

Good goal setting

Good goals are specific and a little challenging. Not too challenging though, because the hardest thing about making a change is often sticking with it. So it’s important to keep your goals realistic. You can alter them at any time if you find they’re too difficult or too easy.

You can set goals for:

1. Number of alcohol free days a week
2. The total amount of alcohol you drink
3. The number of calories you get from alcohol
4. The money you spend on alcohol.

We suggest you start with a couple and take it from there.

Setting a plan for action

Achieving a goal is easier when you’ve got a plan. Planning helps you manage situations when you may be tempted to drink a bit too much.

	For example, how might you plan to avoid having that drink that takes you over the edge? How might you plan for drinking nothing at all for a few days each week?
**About alcohol -> Can't stop drinking**	If you find it difficult to cut down your drinking, or think you may have a problem with your drinking<br>• The NHS has services that might help [link to NHS alcohol services]<br>• Or try alcoholics anonymous [link to AA web site]
**App settings -> Reminders**	It's a good idea to set this for the morning, at a time you'll regularly be able to fill it in, and when your memory is fresh.<br>[Clock dial]<br>We'll help you keep a drinking diary<br>[Reminder on/off] (defaults to on)<br>If this is on we'll prompt you to record your alcohol consumption each day.
**About the app**	**Contact**<br>We would be delighted to help if you've got any questions about the app or the study<br>[David Crane]<br>[Claire Garnett]<br>[Susan Michie] (Principal Investigator)<br>*(Users can tap a name to email the person concerned)*

The team

If you use this app to help you drink less alcohol you're in good hands.

The app has been developed by a highly qualified team of experts based at University College London, with support from leading researchers from other universities. Professor Susan Michie and Professor Robert West both have extensive experience in helping people achieve behaviour change goals. Dr Jamie Brown led a successful project called StopAdvisor to help smokers quit and runs ongoing studies about alcohol use. David Crane has developed a very popular app called [Smoke Free] and Claire Garnett comes to the project as a psychologist with a Master of Science Degree from University College London.

We've developed this app to study whether it helps people drink less alcohol so we can learn how to do this better. We will publish our findings so that others can benefit. So by using the app you're not only helping yourself but are helping science too. Thank you.

This research was funded by the UK Centre for Tobacco and Alcohol Studies (UKCTAS) and the NIHR School for Public Health Research (NIHR SPHR). The views expressed are those of the author(s) and not necessarily those of the NHS, the NIHR or the Department of Health. UKCTAS is a UKCRC Public Health Research Centre of Excellence. Funding from the Medical Research Council, British Heart Foundation, Cancer Research UK, Economic and Social Research

Council and the National Institute for Health Research under the auspices of the UK Clinical Research Collaboration, is gratefully acknowledged. Dave's PhD studentship is funded by NIHR SPHR, Claire's is funded by UKCTAS.

The study and you

(same as for information sheet and consent form)

Privacy policy

All your information will be stored anonymously and securely. You are not obliged to take part in this study and you should not feel coerced. If you choose to withdraw you may do so without disadvantage to yourself and without any obligation to give a reason. To withdraw please got to the Help tab of the app and choose "Opt out of the experiment".

Please feel free to ask us any questions on support@drinklessalcohol.com.

Opt out

Please confirm you wish to opt-out of the experiment

[Opt-out / Cancel]

Rate this app

(links to Apple app store)

Full content specification for intervention modules of the Drink Less app

Normative feedback

Screen	Text	Additional information
Comparison questions	How do you think your drinking compares with others in the UK?<br><br>• Average or lower<br>• High<br>• Top 10%<br>• Lowest 10%<br>*Very low*<br>*Low*<br>*Low-average*<br>*Average (middle 20%)*<br>*High-average*<br>*High*<br>*Very-high*<br>*Top 10%*	Only first four terms shown around the gauge to prevent screen looking cluttered (items in italics not placed around the gauge).<br><br>Every option appears under the gauge whenever the needle is pointing to that bracket.
	How do you think your drinking compares with other [women/men] aged [16-24/25-34/35-44/45-54/55+]?<br><br>• Average or lower<br>• High<br>• Top 10%<br>• Lowest 10%<br>*Very low*<br>*Low*<br>*Low-average*<br>*Average (middle 20%)*<br>*High-average*<br>*High*<br>*Very-high*<br>*Top 10%*	The comparison question relating to the user's relevant gender and age group will automatically select the relevant option based on the socio-demographic data the user inputted.

**Normative feedback (Intensive)**	**Prepare yourself!**<br><br>We're going to show you how your drinking compares with other people and this might come as a bit of a surprise. If so, don't worry, there are plenty of things you can do about it if you want to, and this app is here to help.<br><br>These data come from a large survey of adults in England called the Alcohol Toolkit Study [link to website http://www.alcoholinengland.info].<br><br>The survey asks adults over 16 years old about their drinking – this includes people who don't drink at all who you may have forgotten about when answering the questions on the previous screen. Remember, we usually socialize with people who drink similar amounts and so assume everyone drinks that much.<br><br>**UK gauge**<br><br>Your drinking is greater than x% of other people in the UK.<br><br>You [over-/under-/correctly] estimated how much other people in the UK drink.<br><br>**UK gauge - drinkers**<br><br>Your drinking is greater than x% of other drinkers in the UK.	The statement about their estimation will be based on their answer to the comparison questions.<br><br>A red needle on the gauge indicates their response to the comparison question. A black needle indicates how their drinking actually compares with the relevant comparison group.<br><br>The scale on the gauge refers to percentiles. The colour of the gauge indicates alcohol-related risk and represents the four quartiles (0-25%- green to yellow; 26-50%- yellow to orange; 51-75%- orange to red; 76-100%- red).<br><br>Minimal text directly above the gauge with three labels (for 0-50% - average or lower; 70-80% - high; 90-100% - top 10%). The text underneath the needle of the gauge specifies the exact percentile the user falls in and how accurate their beliefs about how their drinking compares with others are.<br><br>People infographic:<br><br>People infographic with 20 people (each one

You [over-/under-/correctly] estimated how much other drinkers in the UK consume.	representing 5% of population)
**UK people infographic**<br><br>This means for every 20 people in the UK you're at a greater alcohol-related risk than x of them.	The gauges and people infographic have a toggle at the top of the screen allowing users to choose between 'everyone' and 'drinkers'. The way in which drinkers were defined was stated below the toggle button:
**UK people infographic – drinkers**<br><br>This means for every 20 drinkers in the UK you're at a greater alcohol-related risk than x of them.	"Drinkers includes anyone who has had a drink in the last year, even if that was just one!"
**Age/gender gauge**<br><br>Your drinking is greater than x% of other [women/men] aged [16-24/25-34/35-44/45-54/55+].<br><br>You [over-/under-/correctly] estimated how much other [women/men] aged [16-24/25-34/35-44/45-54/55+] drink.	
**Age/gender gauge - drinkers**<br><br>Your drinking is greater than x% of other [women/men] aged [16-24/25-34/35-44/45-54/55+] who drink.<br><br>You [over-/under-/correctly] estimated how much other [women/men] who drink	

	aged [16-24/25-34/35-44/45-54/55+] consume.	
	**Age/gender people infographic**	
	This means for every 20 [women/men] aged [16-24/25-34/35-44/45-54/55+] you’re at a greater alcohol-related risk than x of them.	
	**Age/gender people infographic – drinkers**	
	This means for every 20 [women/men] aged [16-24/25-34/35-44/45-54/55+] who drink you’re at a greater alcohol-related risk than x of them.	
	As we said before, don’t worry if this was a bit of a surprise; it is very common for people to underestimate their drinking. We’ve created this app to help you drink a bit less. So let’s go ahead and get started.	
**Information button (i) (Enhanced)**	This screen lets you know how your drinking compares with other people.	The information button was requested by users to explain the normative feedback screens.
	The red needle on the gauge shows what you have said and the black needle shows how your drinking actually compares.	‘i’ icon on the top right of the screen when showing the normative feedback
	On the infographic, the people highlighted are at greater alcohol-related risk than you.	

**Brief advice (Minimal)**	Drinking too much can put you at an increased risk of a number of things including…<br><br>• Memory loss<br>• Relationship problems<br>• Depression<br>• Impotence<br>• Injury<br>• High blood pressure<br>• Liver disease<br>• Cancer<br>• Weight gain	
**Social norms information (*below average drinking*)**	**UK gauge**<br><br>Your drinking is average or lower than other people in the UK.	A black needle always indicates 50% so the users are never told their drinking levels are below average.
	You [over-/under-/correctly] estimated how much other people in the UK drink.<br><br>**UK people infographic**<br><br>X out of 20 people in the UK drink alcohol once a week or less.	The text below the gauge does not inform the user of their specific percentile. Instead informs the user that "Your level of drinking is average or lower" and how accurate their estimation was.
	**Age/gender gauge**<br><br>Your drinking is average or lower than other [women/men] aged [16-24/25-34/35-44/45-54/55+].	

You [over-/under-/correctly] estimated how much other [women/men] aged [16-24/25-34/35-44/45-54/55+] drink.

Age/gender people infographic

X out of 20 [men/women] aged [16-24/25-34/35-44/45-54/55+] drink alcohol once a week or less.

**Question assessing helpfulness**	If you want to look at these screens again, you can find it in the progress menu.

Was this information helpful?

Yes

No

Cognitive bias re-training module

Screen	Text	Additional information
**Yes please, no thanks (Enhanced)**	This game aims to help you re-train your mind so you get more used to saying “No thanks” to alcohol when you choose to.<br><br>Read the instructions below or start playing now and pick it up as you go.<br><br>*Links to:*<br><br>[Play]<br><br>[Instructions]<br><br>[Previous scores]<br><br>[How re-training your mind works]	The link to previous scores appears after the game has been played twice.
**Yes please, no thanks (Minimal)**	*Links to:*<br><br>[Play]<br><br>[Instructions]<br><br>[Previous scores]	
**Instructions**	**Screen 1**<br><br>You will see pictures in either landscape (short and wide) or portrait (long and think). The key thing to remember is that it’s the shape of the box, not the image itself, which matters.<br><br>When you see a {landscape} picture use your finger to pull it towards you (i.e. down). This is like saying “Yes	

please". When you see a {portrait} picture use your finger to push it away from you (i.e. up). This is like saying "No, thanks".

You will have 60 seconds to see how many pictures you can sort. Quick is good but remember, the quicker you go, the more errors you might make. You get a point for every one you get right but lose two if you get it wrong.

Screen 2

Say "No thanks" to [landscape] pictures by pushing them away from you (animation of pictures being pushed away)

Screen 3

Say "Yes please" to [portrait] pictures by pulling them towards you (animation of pictures being pulled towards the bottom of screen)

Link to

[Play now!] (which takes the user straight to the game)

**Feedback**	• Could do better! You scored [0] points.<br>• Good! You scored [2] points.<br>• You have finished the game. Remember practice makes perfect. Come back and play again to see if you are getting any faster.	Screen showed only after user completed the game.

On 2nd attempt…

- Amazing! You scored x points. You beat your personal best!
- You have finished the game. Do come back and play again to see if you are getting any faster!

On 3rd attempt

- Good! You scored 3 points. You have finished the game. Do come back and play again to see if you are getting any faster!

**How re-training your mind works (Enhanced)**	Research has shown that we sometimes drink but don't know why this is. This is like an unconscious impulse to say "yes" to the idea of drinking. One way to help you stick to a decision to drink less is to reduce the power of that impulse, and a number of studies have shown this to work.
	This game has been designed to help you do just that, and be a bit of fun too.
	If you'd like to read more about the theories and evidence showing how this works, please visit Help > References.
**Information button (i) (Enhanced)**	This game is a fun way to re-train your mind so it gets more used to saying "No thanks" to alcohol.
	Follow the instructions and use your finger to either pull the picture towards you (swipe down) or push it away (swipe up).

	Images are used from the Amsterdam Beverage Picture Set. To find out more about their licensing please visit drinklessalcohol.com
**Information button (i) (Minimal)**	This is meant to be a fun and engaging game.<br><br>Follow the instructions and use your finger to either pull the picture towards you (swipe down) or push it away (swipe up).<br><br>Images are used from the Amsterdam Beverage Picture Set. To find out more about their licensing please visit drinklessalcohol.com

Self-monitoring & Feedback module

Screen	Text
**Dashboard (Enhanced)**	Graph of alcohol consumption in units since the app was downloaded<br>Information about calories consumed from alcohol and money spent on alcohol<br><br>*We suggest*<br>• Logging your drinks each day (disappears when drink diary completed, reappears next day)<br>• Random link to other intervention module<br><br>*Your achievements*<br>• Lists number of consecutive days the drinking diary had been completed<br>• Provides link to goal feedback for each goal set<br><br>*Your active goals*<br>• Provides summary information about performance against goals for the current week: Title of goal, So far x, ends in y days<br><br>*Quick links*<br>• Your hangover and you<br>• Your drinking calendar<br>• Drinking guidelines
	**Information button (i)**<br><br>The dashboard gives you an overview of how much you've drunk, what you've achieved and the goals you've set.<br><br>Your unit consumption is shown weekly. Tap a bar to see a summary of what you got through that week. Tap the words Calories or Money to see how many calories you've consumed in alcohol and how much you've spent on it.<br><br>Your Achievements lists how many times in a row you've kept your diary. You don't have to record drinks to increase your streak! Recording alcohol-free days counts too. This also lists your success against your goals for the week just gone. Tap any for more detail.<br><br>'We Suggest' contains a few ideas we have to help you drink less. The only thing we recommend doing daily is keeping your diary. 'Your Goals'

summarises your progress against your goals for this week. Tap to see progress against them to date.

You'll see links to the things we think you'll find most helpful in the 'Quick Links' section.

Dashboard (Minimal)

No graphs displayed

We suggest
Random link to other intervention module

Your active goals
Provides summary information about performance against goals for the current week: Title of goal, date started

Quick links
- Your drinking calendar
- Drinking guidelines

Information button (i)

The dashboard gives you an overview of the goals you've set and some quick links to further information

'We Suggest' contains a few ideas we have to help you drink less. The only thing we recommend doing daily is keeping your diary. 'Your Goals' summarises your progress against your goals for this week.

You'll see links to the two things we think you'll find most helpful in the 'Quick Links' section.

Drink and mood diary (Enhanced)

These questions may help you gain greater understanding of the effects of alcohol. Your scores on heavy drinking days are compared against light/no-drinking days and displayed on the 'Your hangover and you' screen. Try to answer them at the same time each day

What mood are you in right now?
Grumpy Okay Feel good

How productive do you feel?
Not very Middling Bring it on

How clear-headed do you feel?
Not very Middling Clear as a bell

How did you sleep?
Badly Okay Like a baby

Any comments

Any more drinks since the last one recorded on [date]? No/Yes

Did you drink more than you wanted to yesterday? No/Yes

If No:
What helped you achieve your goal?

If yes:
What got in the way?

[Save]

Information button (i)
Each morning we'll ask four questions about your mood, productivity, clarity and sleep quality. The idea is to help you gain a deeper picture of the effects of alcohol, which we do by comparing your scores on heavy drinking days against light/no-drinking days. You can see the results on the "Your hangover and you" screen.

It's a good idea to answer these questions at the same time each day. Doing so will make comparisons between the scores more accurate.

Any comments you make can be found on the Calendar entry for that day.

Answers to the "Did you drink more than you wanted to yesterday?" question can be found on the "Progress > What has and hasn't worked for you" screen.

Don't be surprised when the Add Drinks screen pop ups after you've answered Yes to "Any alcoholic drinks to record?" It's our way of making your life a little easier.

**Drink and mood diary (Minimal)**	Any more drinks to record since the last one recorded on [date]? Yes/No<br><br>[Save]
**Add drinks**	Date

(Available to all users) Alcohol free day [toggle on/off, defaults to off]

User chooses one of:
Beer/Cider/Wine/Fortified Wine/Spirits/Alcopops

Then selects options for each drink
Details

**Type**	**Options**		**Volume**
Beer	Ale<br>**Lager**<br>Stout<br>Craft		Very small bottle (275ml)<br>½ pint (284ml)<br>Small bottle (330ml)<br>Can (440ml)<br>Large bottle (500ml)<br>Large can (500ml)<br>**Pint (568ml)**
Cider	(None)		Very small bottle (275ml)<br>½ pint (284ml)<br>Small bottle (330ml)<br>Can (440ml)<br>Large bottle (500ml)<br>Large can (500ml)<br>**Pint (568ml)**
Wine	**Red**<br>White<br>Rose<br>Sparkling		Small glass (125ml)<br>**Medium glass (175ml)**<br>¼ bottle (187.5ml)<br>Large glass (250ml)<br>1/3 bottle (250ml)<br>½ bottle (375ml)
Fortified wine	Sherry<br>**Port**<br>Madeira<br>Marsala		Glass (150ml)
Spirits	Options<br>Whisky<br>**Vodka**<br>Rum<br>Gin	Add<br>Nothing (neat)<br>Coke<br>Lemonade<br>Lime<br>**Juice**<br>Diet coke<br>Diet lemonade<br>Tonic<br>Ginger beer/ale<br>Soda<br>Other	**Single (25ml)**<br>Double (50ml)<br>Triple (75ml)
Alcopops	None		Small bottle (275ml)<br>**Bottle (330ml)**

Items appear in the order presented in the app. Items in bold were selected by default.

Regulars
A list of drinks users have added as a regular

Recent
List of drinks users have recently added, most recent first

Information button (i)
Tap the drink type or amount to display options for that item.

To edit ABV (that's the alcoholic strength of your drink) you'll need to tap on the figure itself. It's bit clunky, we know. But we've found it the easiest way to let you set the ABV precisely.

Price per drink works in a similar way, tap the figure to enter or edit.

It probably won't surprise you to learn you can adjust the quantity by tapping the + or - button

To save time next time you can add this as a regular. Regulars are stored in the Regulars tab underneath all the icons of drinks.

Goal feedback (Self-monitoring and Feedback Enhanced)

Last week

Goal: [Goal name]

Last ended: [Date]

Goal record: (e.g. alcohol free days: 3)

Icon	Represents
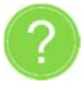	Goal exceeded by ≥ 20%
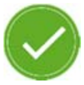	Goal hit
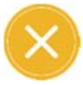	Goal missed
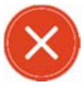	Goal missed by ≥ 20%

Feedback

Criteria	Feedback
Goal exceeded by ≥ 20% once:	Overachiever! Goal smashed. Well done.
Goal exceeded by ≥ 20% twice in a row:	Whoa, there goes that goal again. Twice in a row too. Is this an unusual period or do you think the goal is a bit easy? <u>You can make it harder if you like.</u>
Goal hit; one of (delivered at random):	Get you! Good work on hitting your goal.<br>Congratulations on a great [week/month] of achievement. Feel proud? You should.<br>Goal hit. Good work. You're great.<br>That's your goal got! I'd pat you on the back if I had arms.<br>Well done, you hit your goal. Keep going.
Goal missed; one of (delivered at random):	Didn't quite make this one. Close though. You can do this.<br>Just missed this goal. It's definitely within reach though.<br>Nearly made it. Just need to do a bit more and you'll make it next time.<br>That was close! Won't take much more to get that glorious green tick.<br>Almost! Bit more of a push and you'll get this goal.
Goal missed by ≥ 20%:	You didn't hit your goal this week. No problem, keep going.
Goal missed by ≥ 20% twice in a row:	Looks like you're having a bit of difficulty with this one. Is it an unusual period, or do you think the goal is a bit much of a stretch? <u>You can make it a slightly easier if you like.</u>

Hit rate

Graph of user's progress against the goal since the app was downloaded

You've hit x of your goals to [goal title]

Success rate

Pie chart of proportion of times user has Exceeded, Hit, Nearly hit or Missed the goal

Your longest streak for hitting this goal lasted x weeks

Information button (i)

Last week: This shows how well you did for the week just gone.

Hit rate: How many times you've exceeded, hit, nearly hit, or missed the goal for all previous weeks.

Success rate: Similar to hit rate, this totals the number of times you exceeded, hit, nearly hit or missed the goal since you downloaded the app.

Mood diary feedback (Self-monitoring and Feedback Enhanced)

Mood

Your average mood score on

[Graph comparing mood scores on mornings after no/light drinking days with heavy drinking days]

Alcohol affects many people's mood the day after drinking. Alcohol can make us feel depressed, anxious, irritable or just not ourselves.

Of course, lots of other things can make us feel this way. One way to tell how alcohol affects your mood is to notice the way you feel in the morning and log this in your mood diary.

Productivity

Your average productivity score on

[Graph comparing productivity scores on mornings after no/light drinking days with heavy drinking days]

Alcohol can affect our ability to concentrate, think, and perform tasks; not just when we're drinking, but the morning after too.

Notice how you feel when you awake and for the first few hours after. Log that, and your drinking, in your diary, and we'll show how alcohol affects your feelings of productivity.

Clarity

Your average clarity score on

[Graph comparing clarity scores on mornings after no/light drinking days with heavy drinking days]

Many people say that alcohol makes their thinking more muddled the next day. Thoughts can be harder to form, ideas don't come as easily and problems seem harder to solve.

Keep tabs on how clear headed you feel each morning and log that in your diary. The more often you do this, the more accurate these graphs will be

---

Sleep

Your average sleep score on

[Graph comparing sleep scores on mornings after no/light drinking days with heavy drinking days]

Whilst alcohol can make you sleep more quickly, it can affect the quality of sleep you get. Many people find that alcohol makes them sleep more lightly, wake more often and get less rest.

Alcohol isn't the only thing that affects sleep. Keeping a sleep diary alongside records of your drinking can be a good way to tell how it affects yours.

Information button (i)

These graphs show how your mood, productivity, clarity and sleep are affected by heavy drinking; scores are taken from the answers you gave to the questions in the mood diary.

All the scores on mornings after you've drunk more than [8 men, 6 women] units go in the "Day's after drinking" column. All the scores on mornings after you've drunk less or nothing at all are in the "Days after not drinking" column.

Compare the height of the two columns to see if – and by how much – alcohol affects you the next day.

What has and hasn't worked (Self-monitoring and Feedback Enhanced)

Displays entries from the response to the Mood Diary question: Did you drink more than you wanted to yesterday?

What worked

- List of user entries

What didn't work

- List of user entries

[Text displayed if the user is also in the Action Planning enhanced condition]

If you're having difficulty a plan for how to deal with difficult drinking situations might help [‘plan’ links to the Action Plans screen

Information button (i)

Remember the question in the Mood Diary that asks "Did you drink more than you wanted to yesterday"? This screen lists all the responses you've made there.

Answers to “What helped you achieve your goal?” are in What worked. Answers to “What got in the way?” are in What didn’t work. Reviewing these occasionally might help you achieve that lovely feeling of reaching your goal.

**Calendar (Self-monitoring and Feedback Enhanced)**	**Calendar overview**<br><br>Month (e.g. January)<br><br>[Days in the month underlined with colours representing red for heavy drinking day, orange for light drinking day, green for no drinking day and grey for no record]<br><br>That makes x alcohol free days so far<br><br>---<br><br>**Day when no entry has been made**<br><br>You haven’t recorded any drinks for this day<br><br>Having trouble remembering? It might help to look at your calendar or diary for notes of what you were doing. Or perhaps your text messages or emails can jog your memory<br><br>[Alcohol free day button]<br><br>If alcohol free day button tapped: [Tick image] Keep up the good work!<br><br>---<br><br>**Individual days when drinking has been recorded**<br><br>Today<br><br>Total units x<br><br>[For each recorded drink]<br><br>Type<br>Volume<br>Units/Calories

Information button (i)

Calendar Overview

Tap a date to see your drinking records for that day.

Use the coloured bars to get a quick overview of all your no drinking days, days when you’ve drunk a little, days when… well let’s just say you’ve drunk more than a little, and days you haven’t made an entry for.

Tap the < or > at the top to change the month displayed.

Individual days

If this is empty you can tap the Alcohol Free Day button to celebrate giving your liver a break. Or tap a different part of the screen, or the big + button in the middle of the tab bar below, to enter drinks for that day.

Tap a drink you’ve recorded to edit any part of it.

Swipe it left to delete

Tap the < or > at the top to change the day displayed.

Action Planning module

Screen	Text
**Action Plans (Enhanced)**	One of the best ways of sticking to your goal is to have a plan for managing tricky situations. These IF/Then plans are proven to help people drink less alcohol.<br><br>For example:<br>If I’ve had two drinks and someone offers to buy me another<br>Then I’ll say, ‘No thank you, I’ve got really a busy day tomorrow’<br><br>*Links to:*<br>[Create an action plan]<br>[Your action plans]<br>[Why set an action plan?]<br><br>---<br><br>**Information button (i)**<br>To create an action plan tap ‘Create an action plan’.<br><br>You can see all your action plans by, funnily enough, tapping the ‘Your action plans’ link<br><br>We’ve summarised the evidence for the effectiveness of action plans in ‘Why set an action plan’.
**Create an action plan (Enhanced)**	What might tempt you to drink excessively?<br><br>If (event) [user enters]<br>Then (action) [user enters]<br><br>[Save]<br><br>---<br><br>**Information button (i)**<br>The key thing here is to create both an If and a Then. Here are some examples of what you might write. They might work as is, but are probably better if you personalise them.<br><br>IF I'm out for a drink. THEN I'll drink a glass of water for every glass of alcohol.<br>IF My thought turns to alcohol at five o’clock . THEN I’ll remind myself why I want to drink less.

IF I'm going to pick up a bottle on my way home. THEN I'll remind myself of all the reasons for sticking to my goal.
IF I'm tempted to drink more than I want to. THEN I'll do something on this app first.
IF I'm feeling a strong urge to drink. THEN I'll remember it's a craving and all cravings pass.
IF I'm bored at home and want to drink. THEN I'll complete something on to do list instead.
IF I have too many social occasions in the diary. Then I'll plan in advance how many I'll drink at each one.
IF my friends want to go to the pub. THEN I'll suggest doing something else instead.
IF I am feeling low or anxious. THEN I will try to distract myself with something I love doing.
IF I am out in the pub with friends. THEN I will order small glasses of wine, not large.
IF I'm feeling stressed and want to relax quickly. THEN I'll choose exercise instead.
IF I feel like I want a reward. THEN I'll choose something else I love doing.
IF I want to go to the pub. THEN I'll take my car.

Your action plans (Enhanced)

List of user entered action plans

Information button (i)
Tap any of the rows to see your action plan in full.

Tap Edit in the top right and then any of the rows to make changes to your action plan.

Tap Edit and slide a row right to delete an action plan.

Why set an action plan (Enhanced)

Achieving a goal is easier when you've got a plan. We recommend you plan for tempting situations to help you meet your goal to drink less. Be specific and say IF a certain thing happens THEN you'll do something else.

For example, you could decide: IF I've had two drinks and I'm offered another, THEN I'll say "Thank you, I'll just have an orange juice". It's a good idea to be as specific as you can when creating these plans. Because if they're specific they come to mind more easily right when you need them.

There's plenty of evidence to suggest that IF/THEN plans are effective. So go ahead and set some up for the times you think you'll be tempted to drink more than you want.

Why they work

Here comes the science bit. IF/THEN plans (called implementation intentions) seem to work because having a concrete plan for when in a specific situation means the plan can be performed automatically and without conscious effort. As a result you don't have to rely on willpower, something alcohol is known to weaken.

You might be thinking that implementation intentions are too simple to be effective. However, a review of nearly 100 scientific studies found they "make an important difference to whether or not people achieve their goals".

Action plans (Minimal)

Achieving a goal is easier when you've got a plan. Planning helps you manage situations when you may be tempted to drink a bit too much.

For example, how might you plan to avoid having that drink that takes you over the edge? How might you plan to deal with drinking excessively at parties? How might you plan for drinking nothing at all for a few days each week?

Identity change module

Screen	Text
**Drink + Me (Enhanced)**	Building a picture of yourself that is not based around drinking is an important part of changing your drinking behaviour.<br><br>This section will help you do that.<br><br>*Links to:*<br>[Flipsides of drinking]<br>[Memos]<br>[I am…]
**Flipsides of drinking (Enhanced)**	**Screen 1**<br>We've heard about some of benefits of drinking. But there is often a downside, which you may not have thought about.<br><br>By not drinking too much, you can avoid these downsides and be the person you want to be. Have a flick through our examples that are based on what some people have said.<br><br>To make this section more helpful for you, why don't you add your own examples of what you don't like about drinking too much.<br><br>[Add your own]<br><br>**Screen 2** (ten pairs of images)<br>1. Feel more confident / I'm more likely to become argumentative and aggressive, and be involved in a fight<br>2. Drinking helps me forget problems at work or school / I can become depressed<br>3. Drinking makes socialising easier / I need my friends to look after me and take me home, spoiling their evening<br>4. Alcoholic drinks taste good / I can have a whole days' worth of calories just from drinks and feel too rubbish the following day to go out and exercise<br>5. Drinking makes me feel more romantic / I might have sex with someone who I wouldn't want to if sober<br>6. Drinking helps me think better / My decision making is impaired and I spend far more money than I intended<br>7. Drinking helps calm me down when I'm angry / Too much means I can become aggressive and argumentative, and get into fights

8. Drinking is a nice way to celebrate special occasions / I might have memory lapses and forget the occasion
9. Drinking gives me more confidence in myself / I say something inappropriate that ends up offending someone
10. Feel great and have fun / I feel awful (groggy and tired) the following day

Memos (Enhanced)

This is where you can record messages to yourself to watch in the future. Maybe try saying a tongue twister after you've had some drinks or a message that will persuade you not to drink more than you planned to.
[Add new memo]

Link to:
[Reminders]

Reminders
Here you can set reminders to record memos. It may be helpful to set reminders to record a memo when you're out drinking or the morning after a heavy night. You can also remind yourself to watch one you've already recorded. Maybe at a time you think you're likely to start drinking.

Record memos
[Add Record memo reminder]

Watch memos
[Add Watch memo reminder]

I am… (Enhanced)

Screen 1
This is about thinking about what is important to YOU. And then thinking about how drinking too much can affect whether you live up to them.

Screen 2
Click on the icon to take a photo…

Screen 3
Write a list of the values that are important to you. Or select some of our examples.

- [Important values to me]
- Honest
- Good friend
- Responsible
- Health conscious
- Friendly
- Fun

Screen 4
Sometimes drinking can mean that we behave in ways that do not fit with what we value about ourselves.

Have a think about which of these values you struggle with when you've been drinking too much. Tap to highlight them if you'd like.

Screen 5
Here are some of our examples of values that don't go with getting drunk. Obviously they won't all apply to you though…

- Honest -> Exaggerate stories for 'comic' effect but take it too far
- Good friend -> End up spoiling my friends' night as they need to take care of me once I've had too much to drink
- Responsible -> Spend more money than planned and go over your weekly budget
- Health conscious -> After drinking too much I often have an unhealthy snack, like a kebab, on my way home
- Friendly -> I can get quite argumentative once I have an excessive amount of alcohol
- Fun -> End up half asleep, unable to join in with what's going on

Tap an example to find out more

[Done]

***Would you like to either review your previous entry or start again?
[Review]
[Start Again]
[Cancel]

Information button (i) (Enhanced)

Building a picture of yourself that is not based around drinking is an important part of changing your drinking.

The 'Flipsides of drinking' provide some examples of the negatives about drinking too much, which you may not have thought about. By drinking less, you can avoid these and follow your true values.

You can use 'Memos' to record messages to yourself in the future. This is a chance to record a motivational message about your true self and why you think you should limit your drinking.

The ‘I am’ section is about thinking about what is important to YOU. And then thinking about how drinking too much can affect whether you live up to your ideals.

**Drink + Me (Minimal)**	You are here because you’ve decided that you want to drink less.<br><br>Now, take a moment to imagine yourself as this person who drinks less. What would it mean for you?<br><br>Building up a new identity as someone who does not drink excessively is an important part of drinking less.<br><br>Sometimes the consequences of drinking too much are not what you intended or wanted to happen.<br><br>It can be helpful to think about these negative consequences of drinking too much when you’re trying to drink less.

References

1. Michie S, Whittington C, Hamoudi Z, Zarnani F, Tober G, West R. Identification of behaviour change techniques to reduce excessive alcohol consumption. Addiction [Internet]. 2012 Aug [cited 2014 Mar 13];107(8):1431–40. Available from: http://www.ncbi.nlm.nih.gov/pubmed/22340523
2. Kaner E, Beyer F, Brown J, Crane D, Garnett C, Hickman M, et al. Personalised digital interventions for reducing hazardous and harmful alcohol consumption in community-dwelling populations | Cochrane. 2015 [cited 2015 Apr 26];(1). Available from: http://www.cochrane.org/CD011479/ADDICTN_personalised-digital-interventions-for-reducing-hazardous-and-harmful-alcohol-consumption-in-community-dwelling-populations
3. Garnett C, Crane D, West R, Brown J, Michie S. Identification of Behavior Change Techniques and Engagement Strategies to Design a Smartphone App to Reduce Alcohol Consumption Using a Formal Consensus Method. JMIR mHealth uHealth [Internet]. 2015;3(2):e73. Available from: http://www.ncbi.nlm.nih.gov/pubmed/26123578
4. Crane D, Garnett C, Brown J, West R, Michie S. Behavior change techniques in popular alcohol reduction apps: content analysis. J Med Internet Res [Internet]. 2015;17(5):e118. Available from: http://www.ncbi.nlm.nih.gov/pubmed/25977135
5. Garnett C. Development and evaluation of a theory- and evidence-based smartphone app to help reduce excessive alcohol consumption (unpublished doctoral thesis). University College London; 2016.
6. Crane D. Development and evaluation of a smartphone app to reduce excessive alcohol consumption: Self-regulatory factors. University College London; 2017.
7. Collins SE, Carey KB, Sliwinski MJ. Mailed personalized normative feedback as a brief

intervention for at-risk college drinkers. Journal of studies on alcohol. 2002.

8. Cunningham J a, Neighbors C, Wild TC, Humphreys K. Normative misperceptions about alcohol use in a general population sample of problem drinkers from a large metropolitan city. Alcohol Alcohol. 2012;47(1):63–6.

9. Cunningham JA, Wild TC, Bondy SJ, Lin E. Impact of normative feedback on problem drinkers: a small-area population study. Journal of studies on alcohol. 2001.

10. Kypri K, Langley JD. Perceived social norms and their relation to university student drinking. J Stud Alcohol. 2003;64:829–34.

11. Neighbors C, Larimer MME, Lewis MM a. Targeting misperceptions of descriptive drinking norms: efficacy of a computer-delivered personalized normative feedback intervention. J Consult Clin Psychol [Internet]. 2004 Jun [cited 2014 Sep 11];72(3):434–47. Available from: http://www.ncbi.nlm.nih.gov/pubmed/15279527

12. Wild TC. Personal drinking and sociocultural drinking norms: a representative population study. J Stud Alcohol. 2002 Jul;63(4):469–75.

13. Garnett C, Crane D, West R, Michie S, Brown J, Winstock A. Normative misperceptions about alcohol use in the general population of drinkers: a cross-sectional survey. Addict Behav [Internet]. The Authors; 2015 Mar;42:203–6. Available from: http://dx.doi.org/10.1016/j.addbeh.2014.11.010

14. Baer JS, Stacy a, Larimer M. Biases in the perception of drinking norms among college students. J Stud Alcohol. 1991 Nov;52(6):580–6.

15. Neal DJ, Carey KB. Developing discrepancy within self-regulation theory: Use of personalized normative feedback and personal strivings with heavy-drinking college students. Addict Behav [Internet]. 2004 Feb [cited 2014 Oct 6];29(2):281–97. Available from: http://linkinghub.elsevier.com/retrieve/pii/S0306460303001175

16. Lewis M a, Neighbors C. Social norms approaches using descriptive drinking norms education: a review of the research on personalized normative feedback. J Am Coll

Health [Internet]. 2010 [cited 2013 Dec 5];54(4):213–8. Available from: http://www.pubmedcentral.nih.gov/articlerender.fcgi?artid=2459316&tool=pmcentrez&rendertype=abstract

17. Elster J. Social Norms and Economic Theory. The Journal of Economic Perspectives. 1989. p. 99–117.

18. Miller DT, McFarland C. When social comparison goes awry: The case of pluralistic ignorance. Social comparison: Contemporary theory and research. 1991. p. 287–313.

19. Bechara A. Decision making, impulse control and loss of willpower to resist drugs: a neurocognitive perspective. Nat Neurosci. Nature Publishing Group; 2005 Nov;8(11):1458–63.

20. Strack F, Deutsch R. Reflective and impulsive determinants of social behavior. Pers Soc Psychol Rev. 2004 Jan;8(3):220–47.

21. Wiers RW, Bartholow BD, van den Wildenberg E, Thush C, Engels RCME, Sher KJ, et al. Automatic and controlled processes and the development of addictive behaviors in adolescents: a review and a model. Pharmacol Biochem Behav. 2007 Feb;86(2):263–83.

22. Bowley C, Faricy C, Hegarty B, J Johnstone S, L Smith J, J Kelly P, et al. The effects of inhibitory control training on alcohol consumption, implicit alcohol-related cognitions and brain electrical activity. Int J Psychophysiol. Elsevier B.V.; 2013 Sep;89(3):342–8.

23. Reich RR, Below MC, Goldman MS. Explicit and implicit measures of expectancy and related alcohol cognitions: a meta-analytic comparison. Psychol Addict Behav. NIH Public Access; 2010 Mar;24(1):13–25.

24. Rooke SE, Hine DW, Thorsteinsson EB. Implicit cognition and substance use: a meta-analysis. Addict Behav. 2008 Oct;33(10):1314–28.

25. Thush C, Wiers RW, Moerbeek M, Ames SL, Grenard JL, Sussman S, et al. Influence of motivational interviewing on explicit and implicit alcohol-related cognition and

alcohol use in at-risk adolescents. Psychol Addict Behav. 2009 Mar;23(1):146–51.

26. Wiers RW, van de Luitgaarden J, van den Wildenberg E, Smulders FTY. Challenging implicit and explicit alcohol-related cognitions in young heavy drinkers. Addiction. 2005 Jun;100(6):806–19.

27. Field M, Duka T, Eastwood B, Child R, Santarcangelo M, Gayton M. Experimental manipulation of attentional biases in heavy drinkers: do the effects generalise? Psychopharmacology (Berl). 2007 Jul;192(4):593–608.

28. Schoenmakers TM, de Bruin M, Lux IFM, Goertz AG, Van Kerkhof DH a T, Wiers RW. Clinical effectiveness of attentional bias modification training in abstinent alcoholic patients. Drug Alcohol Depend. 2010 Jun;109(1–3):30–6.

29. Eberl C, Wiers RW, Pawelczack S, Rinck M, Becker ES, Lindenmeyer J. Approach bias modification in alcohol dependence: do clinical effects replicate and for whom does it work best? Dev Cogn Neurosci. Elsevier Ltd; 2013 Apr;4:38–51.

30. Wiers RW, Eberl C, Rinck M, Becker ES, Lindenmeyer J. Retraining automatic action tendencies changes alcoholic patients' approach bias for alcohol and improves treatment outcome. Psychol Sci. 2011 Apr;22(4):490–7.

31. Wiers RW, Rinck M, Kordts R, Houben K, Strack F. Retraining automatic action-tendencies to approach alcohol in hazardous drinkers. Addiction. 2010 Feb;105(2):279–87.

32. Houben K, Havermans RC, Nederkoorn C, Jansen A. Beer à no-go: learning to stop responding to alcohol cues reduces alcohol intake via reduced affective associations rather than increased response inhibition. Addiction. 2012 Jul;107(7):1280–7.

33. Houben K, Nederkoorn C, Wiers RW, Jansen A. Resisting temptation: decreasing alcohol-related affect and drinking behavior by training response inhibition. Drug Alcohol Depend. 2011 Jul;116(1–3):132–6.

34. Schoenmakers T, Wiers RW, Jones BT, Bruce G, Jansen ATM. Attentional re-training

decreases attentional bias in heavy drinkers without generalization. Addiction. 2007 Mar;102(3):399–405.

35. National Institute for Clinical Excellence. Behaviour change: individual approaches. 2014.

36. Boutelle KN, Kirschenbaum DS. Further support for consistent self-monitoring as a vital component of successful weight control. Obes Res. 1998;6(3):219–24.

37. Butryn ML, Phelan S, Hill JO, Wing RR. Consistent Self-monitoring of Weight: A Key Component of Successful Weight Loss Maintenance**. Obesity [Internet]. 2007;15(12):3091–6. Available from: http://www.ncbi.nlm.nih.gov/pubmed/18198319%5Cnhttp://doi.wiley.com/10.1038/oby.2007.368

38. Gaume J, McCambridge J, Bertholet N, Daeppen J-B. Mechanisms of action of brief alcohol interventions remain largely unknown - a narrative review. Front psychiatry [Internet]. 2014 Jan [cited 2014 Sep 22];5(August):108. Available from: http://www.pubmedcentral.nih.gov/articlerender.fcgi?artid=4143721&tool=pmcentrez&rendertype=abstract

39. Jansen JP. Self-monitoring of glucose in type 2 diabetes mellitus: a Bayesian meta-analysis of direct and indirect comparisons. Curr Med Res Opin [Internet]. 2006;22(4):671–81. Available from: http://www.ncbi.nlm.nih.gov/pubmed/16684428

40. Burke LE, Conroy MB, Sereika SM, Elci OU, Styn MA, Acharya SD, et al. The effect of electronic self-monitoring on weight loss and dietary intake: a randomized behavioral weight loss trial. Obes (Silver Spring) [Internet]. Nature Publishing Group; 2011;19(2):338–44. Available from: http://www.pubmedcentral.nih.gov/articlerender.fcgi?artid=3268702&tool=pmcentrez&rendertype=abstract

41. Carver CS, Scheier MF. Control theory: a useful conceptual framework for

personality-social, clinical, and health psychology. Psychol Bull. 1982;92(1):111–35.

42. Dombrowski SU, Sniehotta FF, Avenell A, Johnston M, MacLennan G, Araújo-Soares V. Identifying active ingredients in complex behavioural interventions for obese adults with obesity-related co-morbidities or additional risk factors for co-morbidities: a systematic review. Health Psychol Rev [Internet]. 2012 Mar;6(1):7–32. Available from: http://www.tandfonline.com/doi/abs/10.1080/17437199.2010.513298

43. Ivers N, Jamtvedt G, Flottorp S, Young JM, Odgaard-Jensen J, French SD, et al. Audit and feedback: effects on professional practice and healthcare outcomes. Cochrane database Syst Rev [Internet]. 2012 Jan [cited 2014 Nov 18];6(6):CD000259. Available from: http://www.ncbi.nlm.nih.gov/pubmed/22696318

44. Michie S, Abraham C, Whittington C, McAteer J, Gupta S. Effective techniques in healthy eating and physical activity interventions: a meta-regression. Health Psychol [Internet]. 2009 Nov;28(6):690–701. Available from: http://www.ncbi.nlm.nih.gov/pubmed/19916637

45. Williams SL, French DP. What are the most effective intervention techniques for changing physical activity self-efficacy and physical activity behaviour--and are they the same? Health Educ Res [Internet]. 2011 Apr [cited 2014 Jun 16];26(2):308–22. Available from: http://www.ncbi.nlm.nih.gov/pubmed/21321008

46. Handley M, MacGregor K, Schillinger D, Sharifi C, Wong S, Bodenheimer T. Using Action Plans to Help Primary Care Patients Adopt Healthy Behaviors: A Descriptive Study. J Am Board Fam Med [Internet]. 2006 May 1 [cited 2014 Oct 7];19(3):224–31. Available from: http://www.jabfm.org/content/19/3/224.short

47. Palfai T. Automatic processes in self-regulation: Implications for alcohol interventions. Cogn Behav Pract [Internet]. 2004 Mar [cited 2014 Nov 11];11(2):190–201. Available from: http://www.sciencedirect.com/science/article/pii/S1077722904800300

48. Armitage CJ. Effectiveness of experimenter-provided and self-generated implementation intentions to reduce alcohol consumption in a sample of the general population: a randomized exploratory trial. Heal Psychol [Internet]. 2009 Sep [cited 2014 Nov 27];28(5):545–53. Available from: http://www.ncbi.nlm.nih.gov/pubmed/19751080

49. Hagger MS, Lonsdale A, Koka A, Hein V, Pasi H, Lintunen T, et al. An intervention to reduce alcohol consumption in undergraduate students using implementation intentions and mental simulations: a cross-national study. Int J Behav Med [Internet]. 2012 Mar [cited 2014 Oct 7];19(1):82–96. Available from: http://www.ncbi.nlm.nih.gov/pubmed/21562782

50. Gollwitzer PM. Implementation intentions: Strong effects of simple plans. Am Psychol [Internet]. 1999 Mar;54(7):493–503. Available from: http://www.ncbi.nlm.nih.gov/pubmed/18272806

51. Orbeil S, Hodgldns S, Sheeran P. Implementation Intentions and the Theory of Planned Behavior. Personality and Social Psychology Bulletin. 1997. p. 945–54.

52. Milne S, Orbell S, Sheeran P. Combining motivational and volitional interventions to promote exercise participation: protection motivation theory and implementation intentions. British journal of health psychology. 2002.

53. Piacentini MG, Banister EN. Getting hammered? ...students coping with alcohol. J Consum Behav. 2006;5(April):145–56.

54. Tajfel H, Turner JC. The Social Identity Theory of Intergroup Behavior. The psychology of intergroup behaviour. Chicago: Nelson Hall; 1986. p. 7–22.

55. Oyserman D, Fryberg SA, Yoder N. Identity-based motivation and health. J Pers Soc Psychol. 2007 Dec;93(6):1011–27.

56. Kearney MH, O’Sullivan J. Identity shifts as turning points in health behavior change. West J Nurs Res. 2003 Mar;25(2):134–52.

57. West, Robert, Brown J. Theory of Addiction. John Wiley & Sons.; 2013.

58. Michie S, Johnston M, Abraham C, Lawton R, Parker D, Walker A. Making psychological theory useful for implementing evidence based practice: a consensus approach. Qual Saf Health Care [Internet]. 2005 Feb [cited 2014 May 26];14(1):26–33. Available from: http://www.pubmedcentral.nih.gov/articlerender.fcgi?artid=1743963&tool=pmcentrez&rendertype=abstract

59. West R, Walia A, Hyder N, Shahab L, Michie S. Behavior change techniques used by the English Stop Smoking Services and their associations with short-term quit outcomes. Nicotine Tob Res. 2010 Jul;12(7):742–7.

60. Tombor I, Shahab L, Brown J, West R. Positive smoker identity as a barrier to quitting smoking: findings from a national survey of smokers in England. Drug Alcohol Depend. 2013 Dec;133(2):740–5.

61. Tombor I, Shahab L, Herbec A, Neale J, Michie S, Neale J, et al. Health Psychology Smoker Identity and Its Potential Role in Young Adults ' Smoking Behavior : A Meta-ethnography Smoker Identity and Its Potential Role in Young Adults ' Smoking. Heal Psychol. 2015;
